# Deficiency of the bZIP transcription factors Mafg and Mafk causes misexpression of genes in distinct pathways and results in lens embryonic developmental defects

**DOI:** 10.3389/fcell.2022.981893

**Published:** 2022-08-26

**Authors:** Shaili D. Patel, Deepti Anand, Hozumi Motohashi, Fumiki Katsuoka, Masayuki Yamamoto, Salil A. Lachke

**Affiliations:** ^1^ Department of Biological Sciences, University of Delaware, Newark, DE, United States; ^2^ Department of Gene Expression Regulation, Institute of Development, Aging, and Cancer, Tohoku University, Sendai, Japan; ^3^ Department of Integrative Genomics, Tohoku University Tohoku Medical Megabank Organization, Sendai, Japan; ^4^ Department of Medical Biochemistry, Tohoku University Graduate School of Medicine, Sendai, Japan; ^5^ Center for Bioinformatics and Computational Biology, University of Delaware, Newark, DE, United States

**Keywords:** lens, MAFG, MAFK, transcription, development, epithelium, bZIP transcription factors, eph signaling

## Abstract

Deficiency of the small Maf proteins Mafg and Mafk cause multiple defects, namely, progressive neuronal degeneration, cataract, thrombocytopenia and mid-gestational/perinatal lethality. Previous data shows *Mafg* ^−/−^:*Mafk* ^+/-^ compound knockout (KO) mice exhibit cataracts age 4-months onward. Strikingly, *Mafg* ^−/−^:*Mafk* ^−/−^ double KO mice develop lens defects significantly early in life, during embryogenesis, but the pathobiology of these defects is unknown, and is addressed here. At embryonic day (E)16.5, the epithelium of lens in *Mafg* ^−/−^:*Mafk* ^−/−^ animals appears abnormally multilayered as demonstrated by E-cadherin and nuclear staining. Additionally, *Mafg* ^−/−^:*Mafk* ^−/−^ lenses exhibit abnormal distribution of F-actin near the “fulcrum” region where epithelial cells undergo apical constriction prior to elongation and reorientation as early differentiating fiber cells. To identify the underlying molecular changes, we performed high-throughput RNA-sequencing of E16.5 *Mafg* ^−/−^:*Mafk* ^−/−^ lenses and identified a cohort of differentially expressed genes that were further prioritized using stringent filtering criteria and validated by RT-qPCR. Several key factors associated with the cytoskeleton, cell cycle or extracellular matrix (e.g., *Cdk1*, *Cdkn1c*, *Camsap1*, *Col3a1*, *Map3k12*, *Sipa1l1*) were mis-expressed in *Mafg* ^−/−^:*Mafk* ^−/−^ lenses. Further, the congenital cataract-linked extracellular matrix peroxidase *Pxdn* was significantly overexpressed in *Mafg* ^−/−^:*Mafk* ^−/−^ lenses, which may cause abnormal cell morphology. These data also identified the ephrin signaling receptor *Epha5* to be reduced in *Mafg* ^−/−^:*Mafk* ^−/−^ lenses. This likely contributes to the *Mafg* ^−/−^:*Mafk* ^−/−^ multilayered lens epithelium pathology, as loss of an ephrin ligand, Efna5 (ephrin-A5), causes similar lens defects. Together, these findings uncover a novel early function of Mafg and Mafk in lens development and identify their new downstream regulatory relationships with key cellular factors.

## Introduction

Lens development and homeostasis is important for the establishment and maintenance of its transparency, the perturbation of which causes cataract—defined as opacification of the lens (Lachke and Maas, 2010; Cvekl and Zhang, 2017; Shiels and Hejtmancik, 2019). Depending on its early or late onset in life, cataract is classified as congenital/pediatric or age-related. Deficiency or alterations in several genes and genomic loci have been associated with both congenital and age-related cataract (Hammond et al., 2000, 2001; Congdon et al., 2005; Shiels and Hejtmancik, 2019, 2021; Choquet et al., 2021; Lachke, 2022). An estimated 8–25% of congenital/pediatric cataract are hereditary, suggesting the perturbation of the lens developmental pathways (Haargaard et al., 2004; Yi et al., 2011; Berry et al., 2020). Thus, defining the genetic pathways controlling lens development and/or homeostasis is important for fully understanding the factors contributing toward lens defects and cataract pathobiology. Thus far, the function of several transcriptional factors and post-transcriptional regulators in lens development has been characterized (Dash et al., 2016; Anand and Lachke, 2017; Cvekl and Zhang, 2017; Lachke, 2022).

The *MAF* (musculoaponeurotic fibrosarcoma) gene family encodes basic leucine zipper transcription factors (TFs) and is classified into two subgroups, namely “small” and “large” MAF proteins (Blank, 2008; Kannan et al., 2012; Katsuoka and Yamamoto, 2016). While both small and large MAF proteins contain DNA-binding domains called the Basic Region (BR) domain, small MAFs lack the transactivation domain that is present in large MAFs. Indeed, mutations or deficiency in the large-MAF subgroup gene *MAF* (also known as c-MAF) causes congenital cataract in humans and animal models (Kawauchi et al., 1999; Kim et al., 1999; Ring et al., 2000; Jamieson et al., 2002; Anand et al., 2018a). On the other hand, deficiencies of the small Maf TFs Mafg (OMIM: 602020) and Mafk (OMIM: 600197) in specific combination of knockout alleles are linked to cataract (Agrawal et al., 2015), in addition to other developmental defects such as progressive neuronal degeneration and thrombocytopenia (Blank, 2008; Kannan et al., 2012; Katsuoka and Yamamoto, 2016). Indeed, while individual germline knockout (KO) of either Mafg or Mafk do not lead to discernable defects in the lens, their compound deletion in mice, specifically as *Mafg* ^*−/−*^:*Mafk* ^*+/-*^, results in cataracts from age 4-months onward (Agrawal et al., 2015). Interestingly, *Mafg* ^*−/−*^:*Mafk* ^*+/-*^ compound KO mice do not exhibit defects in early lens stages.

In contrast, in the present study, we find that Mafg and Mafk double KO (*Mafg* ^−/−^:*Mafk* ^−/−^) mice exhibit severe lens defects starting from early stages of lens embryonic development. We report a detailed characterization of the lens defects in *Mafg* ^−/−^:*Mafk* ^−/−^ double KO mice that has revealed the necessity of these small Maf TFs in formation of a uniform monolayered epithelium in lens development. Further, these data suggest that Mafg and Mafk also have a role in coordinating cytoskeletal events in the early stages of epithelial to fiber cell differentiation. RNA-sequencing (RNA-seq) analysis of embryonic lens revealed misexpression of several key genes, including the extracellular matrix peroxidase Pxdn and the Eph signaling receptor Epha5, thereby providing novel insights into the molecular mechanisms underlying the lens defects in *Mafg* ^−/−^:*Mafk* ^−/−^ double KO mice. Together, these data identify new regulatory relationships between key factors that are associated with the maintenance of a monolayered epithelium and cellular differentiation, and therefore may of significance in non-ocular tissues, in addition to the lens.

## Methods

### Generation of *Mafg*:*Mafk* knockout mice

Double heterozygous *Mafg* ^+/-^:*Mafk* ^+/-^ germline knockout (KO) mice, which were generated in a previous study (Shavit et al., 1998; Onodera et al., 2000), were used to derive double knockout (*Mafg* ^−/−^:*Mafk* ^−/−^) and compound KO (*Mafg* ^−/−^:*Mafk* ^+/-^) mouse strains used in this study. Animals were housed in the Office of Laboratory Animal Medicine (OLAM) at the University of Delaware and experiments involving animals adhered to the Association of Research in Vision and Ophthalmology (ARVO) statement for the use of animals in ophthalmic and vision research and were approved by the Institutional Animal Care and Use Committee (IACUC) (IACUC Protocol No. 1226). Unless otherwise mentioned, mice were maintained on an ICR background. Animals were housed at a temperature range of 20–23°C in 12:12-h light-dark cycles with free access to water and food. *Mafg* ^+/-^:*Mafk* ^+/-^ double heterozygous mice were crossed to *Mafg* ^+/-^:*Mafk* ^−/−^ compound KO to generate various KO allele combinations. Genotyping was performed as previously described (Onodera et al., 2000; Agrawal et al., 2015). Briefly, genotyping was performed on tail-DNA prepared from post-natal or embryonic tissue using a commercial DNA-extraction kit (Qiagen, Catalog#158908 (Cell lysis solution), 158,912 (Protein precipitation solution), 158,916 (DNA hydration solution)) and by using the following primers: *Mafg* WT: Forward 5′-GCA​TGA​CTC​GCC​AGG​AAC​AG-3′, *Mafg* WT: Reverse- 5′-CCC​AAG​CCC​AGC​CTC​TCT​AC-3′, *Mafk* WT: Forward 5′-CCT​ACC​GTT​TCT​GTC​TTT​CCA​G-3′, *Mafk* WT: Reverse 5′-AAT​TCC​TGA​GGA​CAA​AGC​TGA​C-3′, and LacZ: 5′-CCT​GTA​GCC​AGC​TTT​CAT​CAA​C-3’.

### Tissue collection and immunostaining

Pregnant female mice collected from crosses between *Mafg* ^+/-^:*Mafk* ^+/-^ and *Mafg* ^+/-^:*Mafk* ^−/−^ mice were harvested for obtaining embryonic tissue at different stages. Observation of the vaginal plug was considered embryonic day (E) 0.5, and tissues was collected at E12.5, E14.5, and E16.5. Mouse embryonic head tissues were embedded without fixation in Optimal Cutting Temperature (OCT) (Fisher Scientific, Catalog# 14-373-65) and stored at -80°C until downstream applications. Tissue was sectioned to obtain coronal sections of the eye using a Leica CM3050 cryostat (Leica Microsystems, Buffalo Grove, IL, United States). Tissue sections were collected on Colorfrost Plus slides (Fisher Scientific, Hampton, NH, United States, Catalog #12-500-18) at chamber temperature of -18°C, at 12 μm thickness for E12.5 and 14 μm thickness for E14.5 and E16.5. For immunostaining, after thawing and air-drying the slides with sections, they were immediately fixed in 4% Paraformaldehyde (PFA) (Fisher Scientific, Catalog# AC416780010) in 1x phosphate buffer saline (PBS) (Corning, Catalog#21-031-CV) for 20 min at room temperature, which was followed by three washes in 1x PBS (5 min each wash). The slides were blocked for 1.5–2 h in blocking buffer (Table 1) followed by overnight incubation at 4°C with the specific primary antibody at the appropriate concentration in blocking or dilution buffer. The following day, section slides were subjected to three washes in 1x PBS (10 min each wash) and incubated for 1.5 h with secondary antibody at 1:200 dilution in blocking or dilution buffer (Table 1) along with the nuclear counterstain 4’,6-diamidino-2-phenylindole (DAPI, 1:1,000 dilution) (Thermo Fisher Scientific; Catalog# 62,248). The specific conditions for different primary antibodies are listed in Table 1. Slides were washed three times in 1x PBS for 10 min each after the secondary antibody incubation followed in mounting media and sealing with cover slips. Slides were stored at −20°C until they were imaged using a Zeiss LSM 880 confocal configured with Argon/Krypton laser (358 and 561 nm excitation wavelengths) (Carl Zeiss Inc., Göttingen, Germany).

**TABLE 1:** Antibodies and immunostaining conditions.

Target	Fixation	Wash	Permeabili-zation	Blocking buffer	Diln. Buffer	Primary antibody	Catalog	Wash	Secondary antibody	Nuclear staining	Wash
MIP (Aquaporin 0 or Aqp0)	4% PFA for 20 min	1X PBS 5 min, 3x each	NA	10% Horse serum (Abcam, Cambridge, MA) in 1X PBS for 1.5 h at RT	NA	1:200 20 h 4°C	Millipore, #AB3071	1X PBS 10 min, 3x each	Alexa 568 Gt anti Rb 1:200 (Alexa Goat anti Rabbit 568, Thermo Fisher Scientific, Catalog # A-11011); incubated with Nuclear Stain for 1.5 h	DAPI 1:1,000	1X PBS 10 min, 3x each
Beta-catenin	4% PFA for 20 min	1X PBS 5 min, 3x each	NA	5% Chicken serum (Abcam, Cambridge, MA), 0.3% TritonX-100, 2% BSA in 1X PBS for 1.5 h at RT	NA	1:50 16 h 4°C	BD Biosciences #610153	1X PBS 10 min, 3x each	Rhodamine Red-X 568 Ch anti Ms 1:200 (Molecular probes, Eugene Oregon United States, R-6388); incubated with Nuclear Stain for 1.5 h	DAPI 1:1,000	1X PBS 10 min, 3x each
Cdkn1c (p57^Kip2^)	4% PFA for 20 min	1X PBS 5 min, 3x each	0.1% TritonX-100 for 5 min	5% Chicken Serum (Abcam, Cambridge, MA), 0.3% TritonX-100, 2% BSA in 1X PBS for 1.5 h at RT	NA	1:50 18 h 4°C	Santa Cruz Biotechnology #sc-8298	1X PBS 10 min, 3x each	Alexa 568 Gt anti Rb 1:200 (Alexa Goat anti Rabbit 568, Thermo Fisher Scientific, Catalog # A-11011); incubated with Nuclear Stain for 1.5 h	DAPI 1:1,000	1X PBS 10 min, 3x each
E-cadherin	4% PFA for 20 min	1X PBS 5min, 3x each	** ** NA	5% Normal Goat Serum (Abcam, Cambridge, MA), 0.3% TritonX-100 (Sigma, Catalog#T8787), 0.3% BSA in 1X PBS for 2 h at RT	NA	1:100 18 h 4°C	Cell Signaling Technology, #3195S	1X PBS 10 min, 3x each	Alexa 568 Gt anti Rb 1:200 (Alexa Goat anti Rabbit 568, Thermo Fisher Scientific, Catalog # A-11011); incubated with Nuclear Stain for 1.5 h	DAPI 1:1,000	1X PBS 10 min, 3x each
F-actin	4% PFA for 20 min	1X PBS 5 min, 3x each	** ** NA	5% Chicken serum (Abcam, Cambridge, MA), 0.1% TritonX-100 in 1X PBS for 1.5 h at RT	NA	NA	Conjugated	1:40 at 1.5 h s at RT	1:40 at 1.5 h at RT (Thermo Fisher Scientific #A12380, Conjugate); incubated with Nuclear Stain for 1.5 h	DAPI 1:1,000	1X PBS 10 min, 3x each
Foxe3	4% PFA for 20 min	1X PBS 5 min, 3x each	0.3% TritonX-100 for 10 min	5% Chicken Serum (Abcam, Cambridge, MA), 0.3% TritonX-100 in 1X PBS for 1.5 h	NA	1:200 20 h 4°C	Santa Cruz Biotechnology #sc-48162	1X PBS 10 min, 3x each	Alexa 568 Gt anti Rb 1:200 (Alexa Goat anti Rabbit 568, Thermo Fisher Scientific, Catalog # A-11011); incubated with Nuclear Stain for 1.5 h	DAPI 1:1,000	1X PBS 10 min, 3x each
Gamma-crystallin	4% PFA for 20 min	1X PBS 5 min, 3x each	** ** NA	5% Chicken serum (Abcam, Cambridge, MA), 0.3% TritonX-100, 1% BSA in 1X PBS for 1.5 h at RT	NA	1:100 18 h 4°C	Santa Cruz Biotechnology #sc-22415	1X PBS 10 min, 3x each	Alexa 568 Dn anti Gt 1:200 (Alexa Dn anti Gt 568, Thermo Fisher Scientific Catalog # A-11057); incubated with Nuclear Stain for 1.5 h	DAPI 1:1,000	1X PBS 10 min, 3x each
Ki-67	4% PFA for 20 min	1X PBS 5 min, 3x each	0.1% TritonX-100 in 1X PBS for 2 min at RT	5% Normal goat serum (NGS) (Abcam, Cambridge, MA), 0.3% TritonX-100 in 1X PBS for 1.5 h at RT	2% BSA, 0.3% TritonX-100 in 1X PBS	1:100 18 h 4°C	Cell Signaling Technology # 9129S	1X PBS 10 min, 3x each	Alexa 568 Gt anti Rb 1:200 (Alexa Goat anti Rabbit 568, Thermo Fisher Scientific, Catalog # A-11011); incubated with Nuclear Stain for 1.5 h	DAPI 1:1,000	1X PBS 10 min, 3x each

### *In situ* hybridization and histology

*In situ* hybridization was performed as previously described (Lachke et al., 2012b). Briefly, mouse embryonic tissue was isolated and fixed in 4% para-formaldehyde, overnight. The tissue freezing media OCT (Tissue Tek, Torrance, CA) was used to embed and freeze the tissue in an orientation to yield coronal sections (16 µm). Oligomers that included T7 promoter sequence upstream of the gene-specific region were used to amplify cDNA that was used as template in an *in vitro* transcription reaction to prepare an antisense digoxygenin-labeled RNA probe. Frozen tissue was thawed and subjected to the previously described *in situ* protocol and images were imaged using light microscope. For hematoxylin and eosin (H&E) staining, mouse embryonic head tissue were fixed in Pen-Fix (Richard Allan Scientific, Kalamazoo, MI) overnight, followed by dehydration using ethanol, and embedding in paraffin. Sagittal paraffin sections (5 µm) were stained with H&E as previously described (Siddam et al., 2018) and visualized using light microscopy.

### Fluorescence quantification and statistical analysis

Fiji ImageJ software (v1.52P, NIH, Bethesda, MD) was used to quantify the differences in the mean fluorescence signal intensity between control (*Mafg* ^+/-^:*Mafk* ^+/-^) and *Mafg* ^−/−^:*Mafk* ^−/−^ KO lens sections. Images were split into a single channel to measure and quantify the mean fluorescence intensity (Ki67, p57^Kip2^, Phalloidin), counting the average cell number, or counting the average number of nuclei (Ki67, p57^Kip2^) as previously described (Shihan et al., 2021). The background subtraction was performed for normalization after the Threshold application, and the fluorescence intensity was measured in the red channel depending on the criteria for each section in at least three biological replicates for control and *Mafg* ^−/−^:*Mafk* ^−/−^ KO lenses. Finally, all statistics were assessed using either Student’s two sample *t*-test (correct for multiple comparisons using the Holm-Sídák method) or one-way ANOVA. Data are presented as mean ± SE (SEM) and differences were considered significant at *p* ≤ 0.05.

### RNA isolation and quality control

For RNA isolation for downstream assays namely, RNA-sequencing (RNA-Seq) and/or RT-qPCR, embryonic lenses at E16.5 (*n* = 8 lenses per biological replicate; total three biological replicates) were collected from control (*Mafg* ^+/-^:*Mafk* ^+/-^), compound (*Mafg* ^−/−^:*Mafk* ^+/-^) and double KO (*Mafg* ^−/−^:*Mafk* ^−/−^) mice. Total RNA isolation was performed using the mirVana™ RNA isolation kit (Thermo Fisher Scientific, Catalog#AM1560), followed by removal of small molecular weight RNA according to the manufacturer’s instructions. RNA quality was analyzed using fragment analyzer (Advanced Analytical Technologies, AATI FEMTO Pulse) and samples with RNA quality number (RQN) greater than 7.2 were used for library preparation and RNA-seq.

### RNA-sequencing

Lens RNA at E16.5 from control (*Mafg* ^+/-^:*Mafk* ^+/-^), compound (*Mafg* ^−/−^:*Mafk* ^+/-^) and double KO (*Mafg* ^−/−^:*Mafk* ^−/−^) mice was used to generate strand-specific, paired-end 101 bp-length libraries which were sequenced by DNA Link, United States (901 Morena Blvd. Ste 730 San Diego, CA 92117, United States) on a NovaSeq 6000 (San Diego, CA, United States). The processed reads were aligned against *Mus musculus* reference genome (mm39) using HISAT2 (Kim et al., 2015). The aligned reads were assembled using StringTie (Pertea et al., 2015) to obtain transcript-level expression counts. The count file was imported in edgeR package using R-statistical environment (Robinson et al., 2010) to analyze differential gene expression in control, compound and double KO datasets. In edgeR, reads that were lowly expressed, i.e. < 1 count per million in less than two samples were filtered out. The RNA-seq data is deposited in GEO and the accession number is: GSE207853.

### Gene ontology analysis for *Mafg* ^−/−^:*Mafk* ^−/−^ dfferentially expressed genes

The Database for Annotation, Visualization and Integrated Discovery (DAVID, v6 .8) was used for functional annotation by Gene Ontology (GO) categories (Huang et al., 2009). The pathways and GO categories identified were prioritized based on Benjamini corrected significant *p*-values. All GO comparisons were made against the 14 October 2020 release of the Gene Ontology Consortium (GOC) database (Ashburner et al., 2000), specifically KEGG (Kanehisa and Goto, 2000).

### cDNA synthesis and RT-qPCR

The isolated mouse embryonic lens RNA was used for cDNA synthesis using iScript cDNA Synthesis Kit (Bio-Rad, Catalog#1708890) followed by quantitative PCR (RT-qPCR). iScript reaction [5x iScript reaction mixture (4 µL), iScript reverse transcriptase (1 µL), Total RNA Template (300 ng), Nuclease-free H_2_O (to make to total volume 20 µL)] was performed using a custom program [25°C (5 min), 42°C (30 min), 85°C (5 min), 4°C (hold)]. RT-qPCR was performed using cDNA-specific primer sets (Table 2) and a Power SYBR Green kit (Fisher Scientific, Catalog # A25742). The RT-PCR reaction [PowerUp SYBR master mix (12.5 µL), Forward Primer (0.5 µL of 10 µM), Reverse Primer (0.5 µL of 10 µM), cDNA (1 µL of 400 ng), Nuclease-free H_2_O (10.5 µL to bring volume to total 20 µL volume)] using a custom program [95°C (2 min), followed by 40 cycles of 95°C (5 s) followed by 58°C (20 s), and terminal cycle of 95°C (15 s), 60°C (1 min), 95°C (15s) and hold at room temperature] was analyzed on a 7500 Fast PCR system (Applied Biosystems, Foster City, California). Three biological replicates (each biological replicate having two technical replicates) were used for control and KO samples. Fold-change was calculated using the ΔΔCT-method using *Gapdh* as a house-keeping gene and statistical significance was determined using a two-sample Student’s t-test.

**TABLE 2:** Primers used in RT-qPCR assays.

Gene	Forward 5′-3′ primer	Reverse 5′-3′ primer	cDNA Amplicon (bp)
*Camsap1*	TAT​TGC​CCA​GAG​CAG​ATG​AAA	CCT​CAG​AAG​GCG​GAT​GTT​ATA​G	90
*Cdk1*	AAA​GCG​AGG​AAG​AAG​GAG​TG	CCA​TGG​ACA​GGA​ACT​CAA​AGA	144
*Cdkn1c*	TGAAGGACCAGCCTCTCT	TCCTGCGCAGTTCTCTTG	99
*Col3a1*	CCC​TTC​TTC​ATC​CCA​CTC​TTA​TT	GAT​CCT​GAG​TCA​CAG​ACA​CAT​ATT	139
*Epha5*	ACC​TGC​ATC​TGT​GTA​TGT​CTT​C	ACT​GAC​ACT​GGT​GTG​GTT​TC	99
*Gapdh*	GAT​CGT​GGA​AGG​GCT​AAT​GA	GAC​CAC​CTG​GTC​CTC​TGT​G	340
*Hmox1*	GTA​CAC​ATC​CAA​GCC​GAG​AA	TGG​TAC​AAG​GAA​GCC​ATC​AC	98
*Lars2*	GAC​AAG​GAA​GGA​TGT​GGA​GAA​G	GGA​ACA​TGG​AGA​GCA​AGT​AGA​A	110
*Mafk*	TGT​TGT​TCT​TCG​CCG​AGT​C	ACAAGCGCTTCTGCTCTC	88
*Mafg*	GAGGCCCTGCAGAACTTT	AGC​ATC​CGT​CTT​GGA​CTT​TAC	147
*Map3k12*	GAG​TGA​CAA​GAG​CAC​CAA​GAT	GGA​CCA​GAT​GTC​AAC​CTT​CTC	103
*Sipa1l1*	GTC​GGT​GGA​GAG​CTT​CAT​TAG	CCA​TTT​CTT​CGC​AGA​GTC​ATT​TC	108
*Trex1*	CAG​GGA​ATG​GTT​CGA​GGA​AA	TGA​GCA​GGG​TTA​GAA​CAT​CAC	111

## Results

### *Mafg* ^−/−^:*Mafk* ^−/−^ KO mice exhibit abnormal multilayered lens epithelium

**FIGURE 1:**
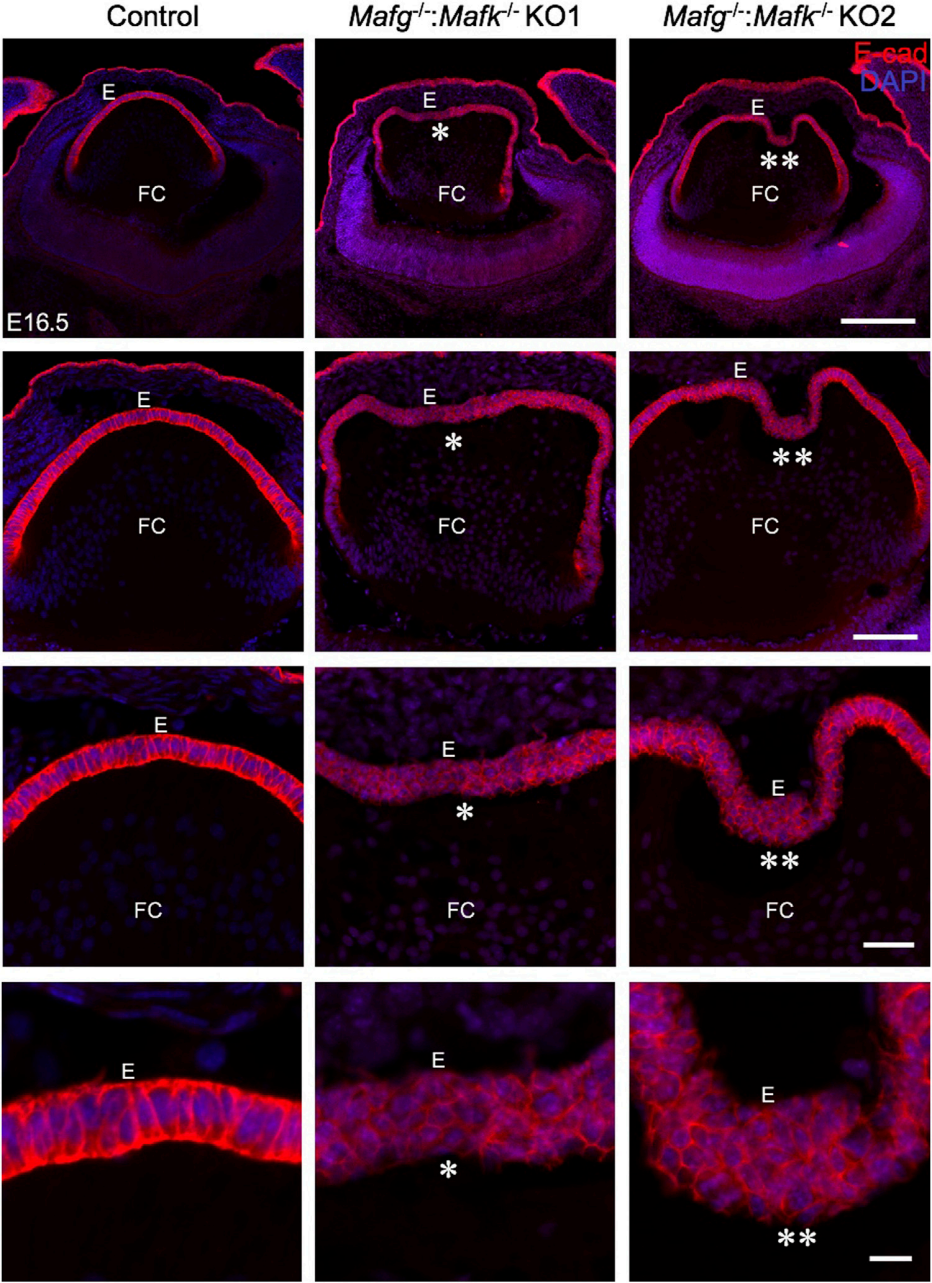
Mafg and Mafk deficiency results in an abnormally multilayered epithelium of the lens. Immunostaining for E-cadherin reveals cellular abnormalities (indicated by asterisks) in the lens epithelium of *Mafg* ^−/−^:*Mafk* ^−/−^ but not control at embryonic day (E)16.5. *Mafg* ^−/−^:*Mafk* ^−/−^KO #1 and KO #2 are shown as representative of the severity of epithelial defects. Sections are co-stained with DAPI for visualization of nuclei. Higher magnification images in row 2 (20X), row 3 (40X), and row 4 (63X) demonstrate the variable extent of multilayered epithelium in the *Mafg* ^−/−^:*Mafk* ^−/−^ lenses compared to control. Abbreviation: E, Epithelium of the lens; FC, Fiber cells of the lens. Scale bar for Row 1, 10 μm; Row 2, 20 μm; Row 3, 10 μm; Row 4, 8 μm.

**FIGURE 2:**
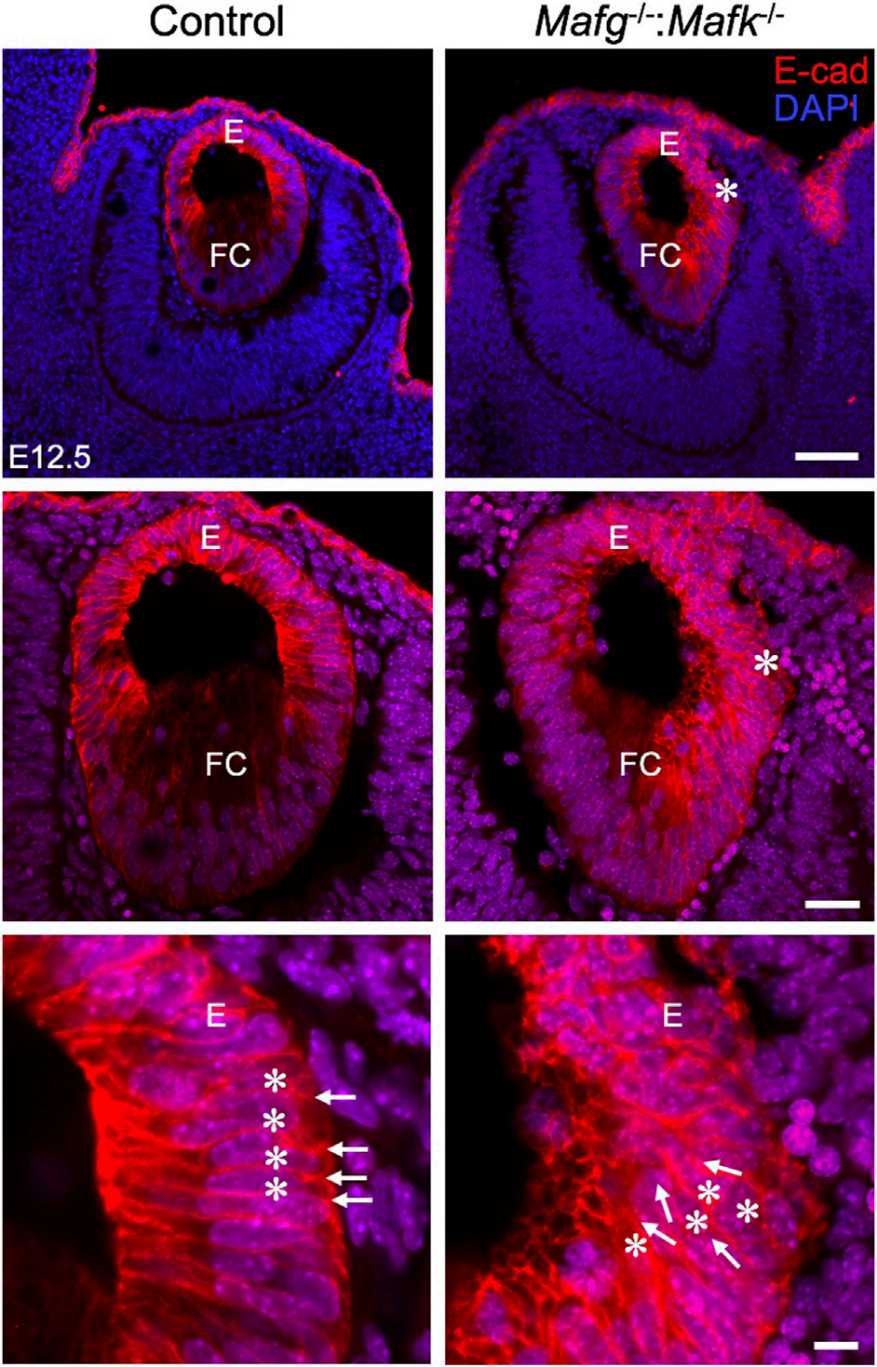
*Mafg* ^−/−^:*Mafk* ^−/−^ mice exhibit abnormalities in the lens epithelium at E12.5. Immunostaining for E-Cadherin and co-staining with DAPI shows that early in lens development, at stage E12.5, *Mafg* ^−/−^:*Mafk* ^−/−^ lens exhibits regions of disorganization in the epithelium (asterisks in row 1, row 2) compared to control. In row 3, cell boundaries (arrows) revealed by E-cadherin staining and nuclei stained by DAPI (asterisks) appear in a uniform manner in the control, but not in *Mafg* ^−/−^:*Mafk* ^−/−^ lens, wherein they appear disorganized. Abbreviation: E, Epithelium of the lens; FC, Fiber cells of the lens. Scale bar for Row 1, 20 μm; Row 2, 10 μm; Row 3, 8 μm.

iSyTE (integrated Systems Tool for Eye gene discovery) expression analysis has indicated that Mafg and Mafk are significantly expressed in mouse lens embryonic development (Supplementary Figure S1A). The expression of *Mafg* in mouse embryonic lens development is confirmed by *in situ* hybridization (Supplementary Figure S1B). Further, examination of previous generated RNA-seq data from isolated epithelium and fiber cells at E14.5, E16.5, E18.5 and P0 (Zhao et al., 2018) shows that *Mafg* and *Mafk* are both expressed in the epithelium and fiber cells (Supplementary Figure S1C). This analysis shows that as relative expression of *Mafg* decreases, that of *Mafk* increases, in progressive stages of development. Together, these data suggesting a role for Mafg and Mafk in lens embryonic development. Therefore, to examine the impact of Mafg and Mafk deficiency on lens development, we generated *Mafg* ^−/−^:*Mafk* ^−/−^ double KO mice and first characterized the lens tissue with marker analysis. Immunostaining for the epithelial marker, E-cadherin (also known as Cdh1), demonstrated profound abnormalities in the appearance of the epithelium in *Mafg* ^−/−^:*Mafk* ^−/−^ lenses. At E16.5, control lenses exhibit uniform E-cadherin protein expression and localization in the epithelium, which appears monolayered, as also suggested by nuclei stained with DAPI (Figure 1). In contrast, the *Mafg* ^−/−^:*Mafk* ^−/−^ lenses exhibit nuclear staining that indicates multilayered epithelium wherein the E-cadherin protein expression pattern also appears abnormal. E-cadherin staining reveals that *Mafg* ^−/−^:*Mafk* ^−/−^ epithelial cells appear irregularly shaped and aggregated compared to control. While this defect is observed in the vast majority of *Mafg* ^−/−^:*Mafk* ^−/−^ lenses (92%, *n* = 12), the extent of the multilayered epithelium cellular abnormalities and cell shape changes varies between individual embryos (Figure 1). In *Mafg* ^−/−^:*Mafk* ^−/−^ lenses that exhibit a comparatively milder defect, the abnormal multilayered cellular region is flanked by normal-appearing mononucleated epithelium. To identify the onset of these defects, we examined embryos at earlier stages. While less severe when compared to the defects observed at E16.5, the E-cadherin protein staining pattern appears abnormal in *Mafg* ^−/−^:*Mafk* ^−/−^ epithelium at E12.5 (Figure 2) and E14.5 (Supplementary Figure S2). Interestingly, the E12.5 *Mafg* ^−/−^:*Mafk* ^−/−^ epithelium exhibits early indications of multilayer formation of cells (Figure 2), suggesting that the lens defects may initiate at this stage and become progressively severe in development. Additionally, when compared to control lenses, the population of E-cadherin expressing cells appears to extend further toward the posterior region in *Mafg* ^−/−^:*Mafk* ^−/−^ lenses (Figures 1, 2). Care was taken to analyze centrally located sections, avoiding peripheral sections. However, it should be noted that not all *Mafg* ^−/−^:*Mafk* ^−/−^ lenses had an abnormal expansion of epithelial cells in the posterior region of the lens. It should also be noted that no overt changes were observed between control and *Mafg* ^−/−^:*Mafk* ^−/−^ lenses with respect to E-cadherin’s localization within the cells. In contrast to *Mafg* ^−/−^:*Mafk* ^−/−^ animals, *Mafg* ^−/−^:*Mafk* ^+/-^ compound mice did not exhibit such severe lens defects at E16.5 (Supplementary Figure S3), suggesting that the absence of both alleles of Mafg and Mafk is necessary to cause these severe lens defects.

### *Mafg* ^−/−^:*Mafk* ^−/−^ KO mouse lenses have abnormal F-actin distribution in fiber cells

**FIGURE 3:**
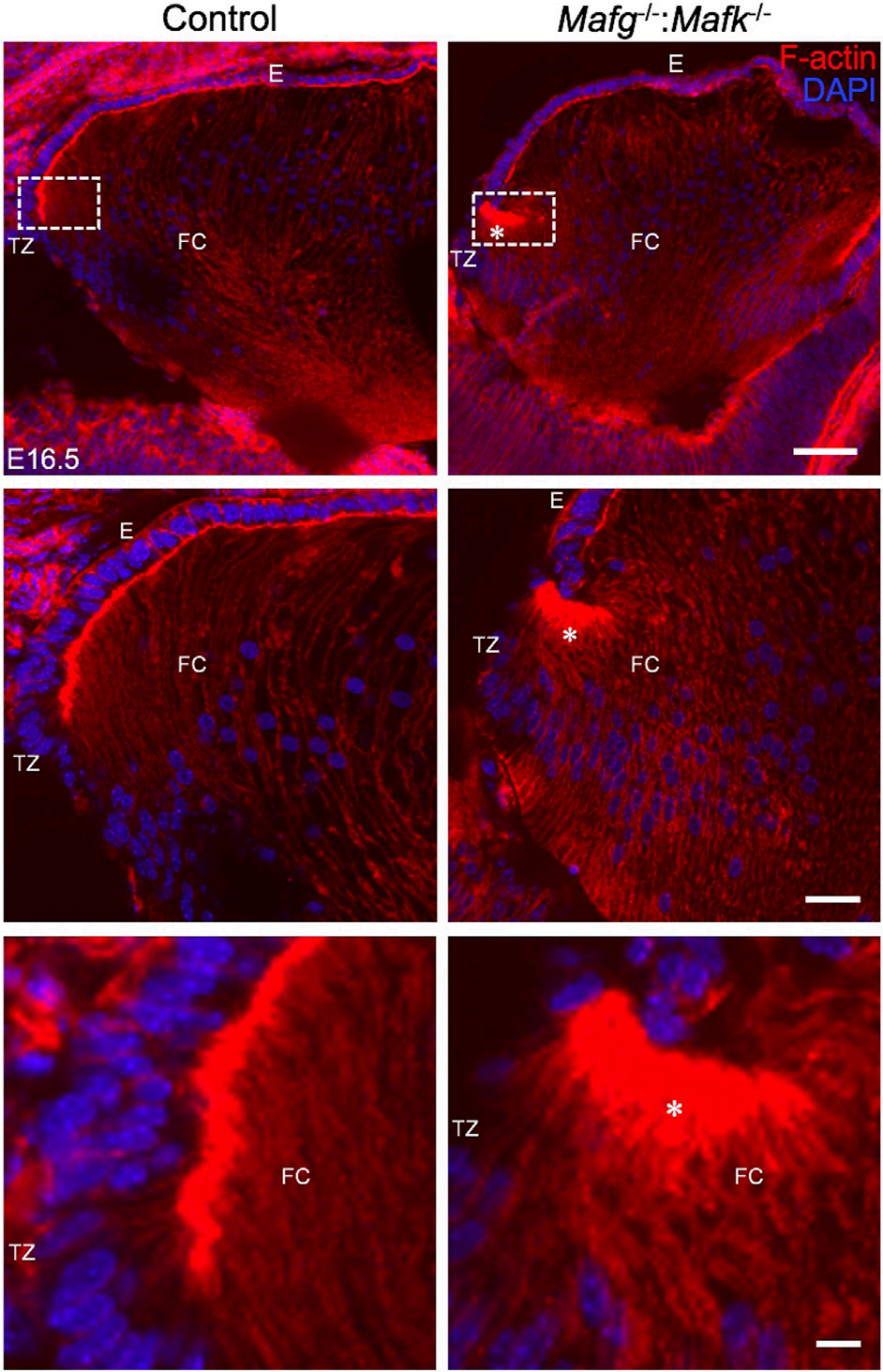
*Mafg* ^−/−^:*Mafk* ^−/−^ mice exhibit abnormal F-actin distribution in differentiating lens fiber cells. Staining with phalloidin for visualizing F-actin and co-staining with DAPI to visualize nuclei demonstrate that E16.5 *Mafg* ^−/−^:*Mafk* ^−/−^ mice exhibit non-uniform distribution (asterisk) of F-actin at the apical tips of the fiber cells near the transition zone. The area shown in the broken-line box in control and test was used for measurement of fluorescence signal intensity shown in Figure 4B. Abbreviation: E, Epithelium of the lens; FC, Fiber cells of the lens; TZ, Transition zone of the lens. Scale bar for Row 1, 20 μm; Row 2, 10 μm; Row 3, 8 μm.

**FIGURE 4:**
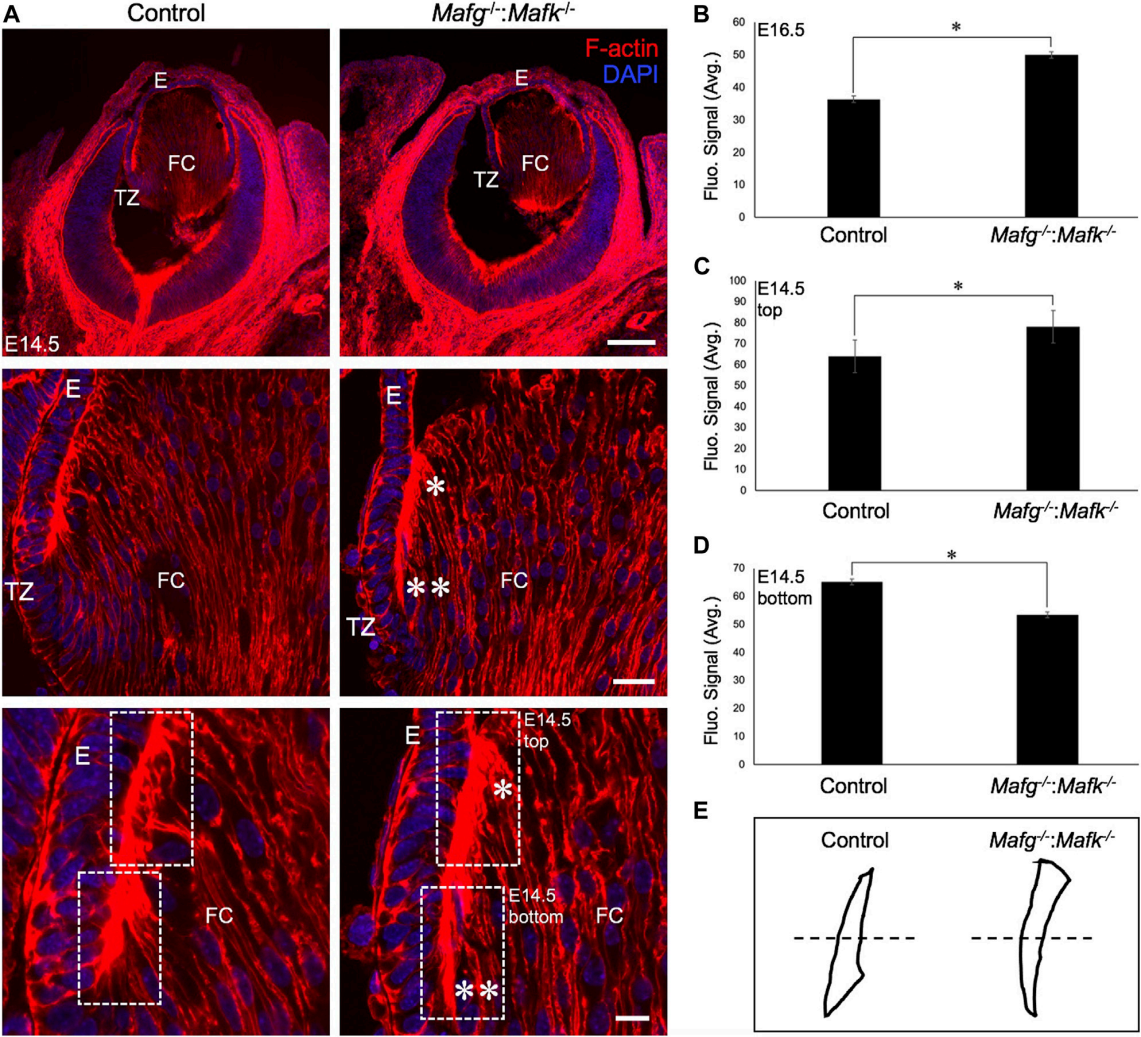
The F-actin distribution defects are detected in earlier stages of lens development in *Mafg* ^−/−^:*Mafk* ^−/−^ mice. **(A)** Staining with phalloidin for visualizing F-actin and co-staining with DAPI to visualize nuclei demonstrate that *Mafg* ^−/−^:*Mafk* ^−/−^ lenses at stage E14.5 exhibit abnormally reduced F-actin distribution (two asterisks) near the transition zone and the fulcrum region compared to the control. Further, *Mafg* ^−/−^:*Mafk* ^−/−^ lenses exhibit abnormally elevated F-actin distribution (asterisk) in fiber cells that have progressed further in differentiation. **(B)** Measurement of fluorescence intensity signal near the transition zone region of E16.5 *Mafg* ^−/−^:*Mafk* ^−/−^ lenses indicates elevated F-actin levels compared to control. **(C)** Measurement of fluorescence intensity signal in fiber cells progressed further in differentiation (represented by broken-line box termed “top”) of E14.5 *Mafg* ^−/−^:*Mafk* ^−/−^ lenses indicates elevated F-actin levels compared to control. **(D)** Measurement of fluorescence intensity signal in fiber cells near the transition zone and the fulcrum region (represented by broken-line box termed “bottom”) of E14.5 *Mafg* ^−/−^:*Mafk* ^−/−^ lenses indicates reduced F-actin levels compared to control. **(E)** Schematic of the differential distribution of F-actin in the top and bottom region (separated by broken line) of control and *Mafg* ^−/−^:*Mafk* ^−/−^ lenses at E14.5. Statistical significance was determined using a two-sample Student’s *t*-test. Abbreviation: E, Epithelium of the lens; FC, Fiber cells of the lens; TZ, Transition zone of the lens. Scale bar for Row 1, 20 μm; Row 2, 10 μm; Row 3, 8 μm.

We next analyzed *Mafg* ^−/−^:*Mafk* ^−/−^ mice for potential defects in lens fiber cells. Near the equator of the lens, at a region termed the transition zone, cells of the epithelium exit the cell cycle and begin differentiation into fiber cells. At the initial stage of differentiation, as fiber cells begin to migrate toward the interior of the lens, they undergo a sharp re-orientation in their position relative to the epithelium. At the lens equator location termed “the fulcrum” or “modiolus”, the apical regions of epithelial cells constrict to form an anchor point prior to elongation during their differentiation into fiber cells (Zampighi et al., 2000; Sugiyama et al., 2009). As a result, at this location, early fiber cells first begin to get positionally re-oriented so that their apical regions face the apical regions of cells of the epithelium. We sought to examine this region in *Mafg* ^−/−^:*Mafk* ^−/−^ mouse lens. Staining for phalloidin (which stains F-actin) showed that E16.5 *Mafg* ^−/−^:*Mafk* ^−/−^ mice exhibit abnormal F-actin distribution in cells near the fulcrum region of the lens (Figure 3). While all E16.5 *Mafg* ^−/−^:*Mafk* ^−/−^ mice that were examined showed this defect on at least one side of the lens, in about one-third of the animals it was observed on both sides. Further, while in E16.5 control lenses, F-actin appears uniformly distributed at the apical junctions of epithelial and fiber cells, it appears reduced in the region anterior to the fulcrum in *Mafg* ^−/−^:*Mafk* ^−/−^ lenses (Figure 3). This abnormal F-actin staining pattern was also observed earlier in development, at E14.5, in *Mafg* ^−/−^:*Mafk* ^−/−^ lenses (Figure 4A). In control lenses, F-actin staining intensity is highest at the initial junction region of epithelial and differentiating fiber cells. In contrast, in *Mafg* ^−/−^:*Mafk* ^−/−^ lenses, the highest staining intensity of F-actin is observed anterior to this region (Figures 4B–E). It should be noted that the quantitative analysis serves to demonstrate that F-actin levels are abnormally distributed in the area measured and are not necessarily a reflection of change in the total actin levels in the lens. Further, histological analysis shows that while in control, the fulcrum region appears normal, in E16.5 *Mafg* ^−/−^:*Mafk* ^−/−^ mice, the fiber cell organization in this region, and beyond, is abnormal, suggestive of suboptimal interactions with the overlying epithelium (Supplementary Figure S4A). Moreover, in control lens, fiber cells appear to “curve” in the same direction as the overall structure of the lens (Supplementary Figure S4B). In contrast, fiber cells do not follow this natural curvature and their appearance is somewhat sigmoidal in *Mafg* ^−/−^:*Mafk* ^−/−^ mice (Supplementary Figure S4B,C). Together, these data indicate that Mafg and Mafk deficiency results in abnormal abundance of F-actin in distinct regions of early differentiating fiber cells, which in turn impacts their organization in the lens.

### Beta-catenin and gamma crystallin proteins are unaltered in *Mafg* ^−/−^:*Mafk* ^−/−^ KO mouse lenses

Because E-cadherin is known to interact with beta-catenin, and both are implicated in cell-cell adhesion, we next sought to examine whether beta-catenin was altered in *Mafg* ^−/−^:*Mafk* ^−/−^ lenses. Immunostaining for beta-catenin demonstrated no change in the levels of its expression in *Mafg* ^−/−^:*Mafk* ^−/−^ lenses at E14.5 (Supplementary Figure S5A) or E16.5 (Supplementary Figure S5B). However, because beta-catenin is localized to the membrane, these data offer independent validation of these defects and their impact on the lens through the view of the cell membrane in *Mafg* ^−/−^:*Mafk* ^−/−^ mice. These data corroborate that while in control lenses, epithelium and fiber cell organization appears uniform, in *Mafg* ^−/−^:*Mafk* ^−/−^ lenses, cells in these regions appear disorganized at E14.5 (Supplementary Figure S5A’,A’’) and E16.5 (Supplementary Figure S5B’,B’’). At E16.5, the cortical fiber cell nuclei appear different between control and *Mafg* ^−/−^:*Mafk* ^−/−^ lenses. Together with the findings on E-cadherin, these data suggest a general loss of normal epithelium architecture in *Mafg* ^−/−^:*Mafk* ^−/−^ lenses. Furthermore, expression of the fiber cell marker, gamma crystallin, was unaltered in *Mafg* ^−/−^:*Mafk* ^−/−^ lenses at E16.5 or earlier stages (Supplementary Figure S6A,B). Together, these data suggest that while the morphology of lens cells is abnormal, certain aspects of fiber cell gene expression are unaltered in *Mafg* ^−/−^:*Mafk* ^−/−^ lenses.

### *Mafg* ^*−/−*^:*Mafk* ^−/−^ KO mouse lenses exhibit altered transcriptome

**FIGURE 5:**
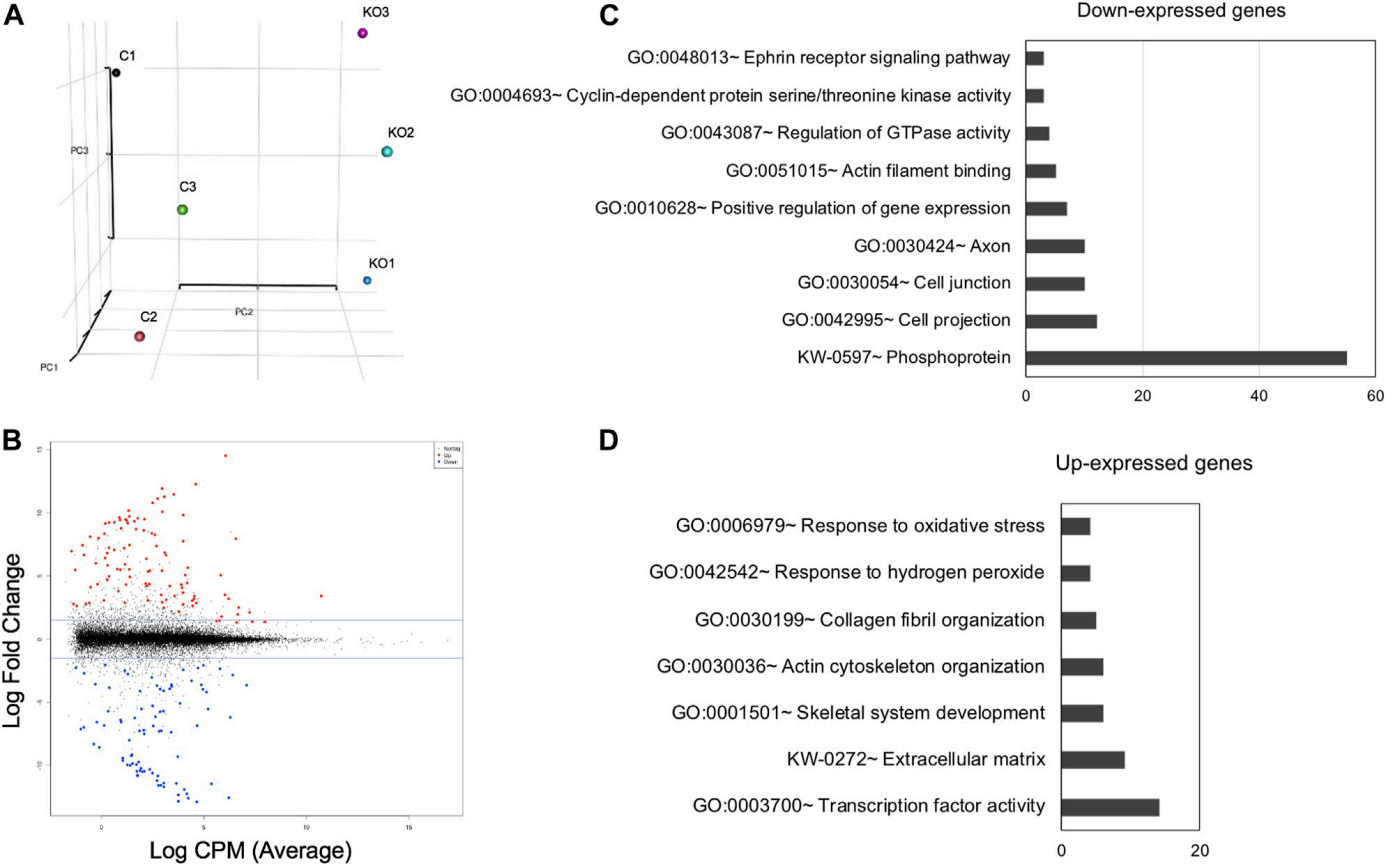
High-throughput RNA-sequencing analysis identifies genes misexpressed in *Mafg* ^−/−^:*Mafk* ^−/−^ lenses. **(A)** Principal Component Analysis (PCA) shows that the RNA-sequencing (RNA-seq) datasets representing *Mafg* ^+/-^:*Mafk* ^+/-^ (control) and *Mafg* ^−/−^:*Mafk* ^−/−^ (double KO) lens cluster based on their genotypes. **(B)** Plot of differentially expressed genes (DEGs) in *Mafg* ^−/−^:*Mafk* ^−/−^ lens compared to control. **(C)** Gene Ontology (GO) and pathway analysis of down-regulated genes in *Mafg* ^−/−^:*Mafk* ^−/−^ lens. **(D)** Gene Ontology (GO) and pathway analysis of up-regulated genes in *Mafg* ^−/−^:*Mafk* ^−/−^ lens.

To identify the specific RNA changes resulting from Mafg and Mafk deficiency, we next took an unbiased genome-wide approach and performed high-throughput RNA-sequencing (RNA-seq) of E16.5 *Mafg* ^−/−^:*Mafk* ^−/−^ lenses. Principle component analysis showed that control and *Mafg* ^−/−^:*Mafk* ^−/−^ lens replicates clustered away from each other (Figure 5). Further, as expected, compared to control, RNA-seq identified *Mafg* and *Mafk* to be severely reduced in *Mafg* ^−/−^:*Mafk* ^−/−^ lenses. Comparative analysis showed that *Mafg* ^−/−^:*Mafk* ^−/−^ exhibit 241 differentially expressed genes (DEGs) (±≥1.5-fold, *p* ≤ 0.05) with 144 being elevated and 97 being reduced in the absence of Mafg and Mafk (Supplementary Table S1).

### Validation of key *Mafg* ^−/−^:*Mafk* ^−/−^ DEGs relevant to lens biology

**FIGURE 6:**
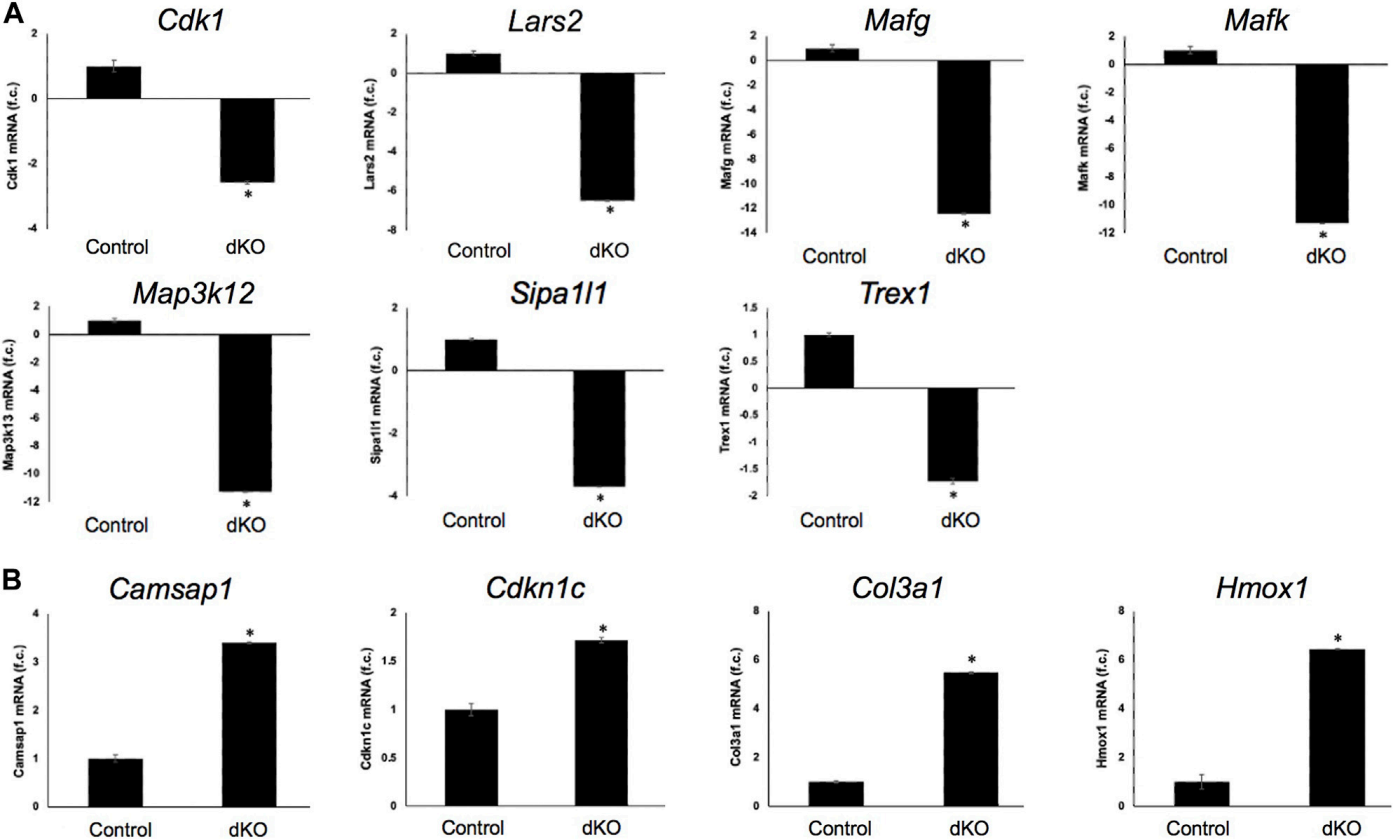
RT-qPCR-based validation of differentially expressed genes in *Mafg* ^−/−^:*Mafk* ^−/−^ lens. Validation by RT-qPCR of several RNA-seq analysis-identified candidate genes that are **(A)** down-regulated and **(B)** upregulated in *Mafg* ^−/−^:*Mafk* ^−/−^ lenses. Fold-change was calculated using the ΔΔCT-method using *Gapdh* as a house-keeping gene and statistical significance was determined using a two-sample Student’s *t*-test. Asterisk indicates *p* ≤ 0.05.

**FIGURE 7:**
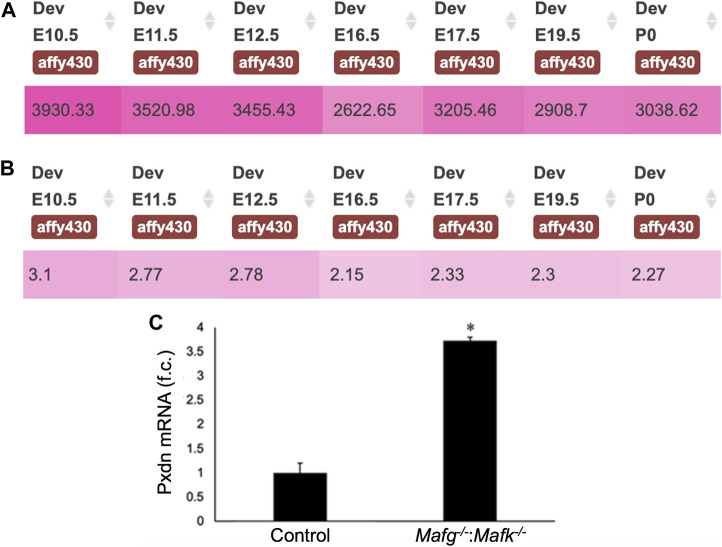
*Pxdn* expression in normal lens development and validation of its abnormally elevated expression in *Mafg* ^−/−^:*Mafk* ^−/−^ lens. **(A)** iSyTE analysis shows that the cataract and ocular defects-linked heme-containing peroxidase enzyme, Pxdn, which is identified by RNA-seq analysis among the DEGs in *Mafg* ^−/−^:*Mafk* ^−/−^ lenses, exhibits robust absolute expression, and **(B)** lens-enriched expression, in various stages of normal mouse embryonic lens development. **(C)** RT-qPCR validation of abnormally elevated expression of *Pxdn* in *Mafg* ^−/−^:*Mafk* ^−/−^ lenses. Numbers in **(A)** represent normalized microarrays fluorescence intensity units as described in Kakrana and coworkers (2018). Fold-change was calculated using the ΔΔCT-method using *Gapdh* as a house-keeping gene and statistical significance was determined using a two-sample Student’s *t*-test. Asterisk indicates *p* ≤ 0.05.

**FIGURE 8:**
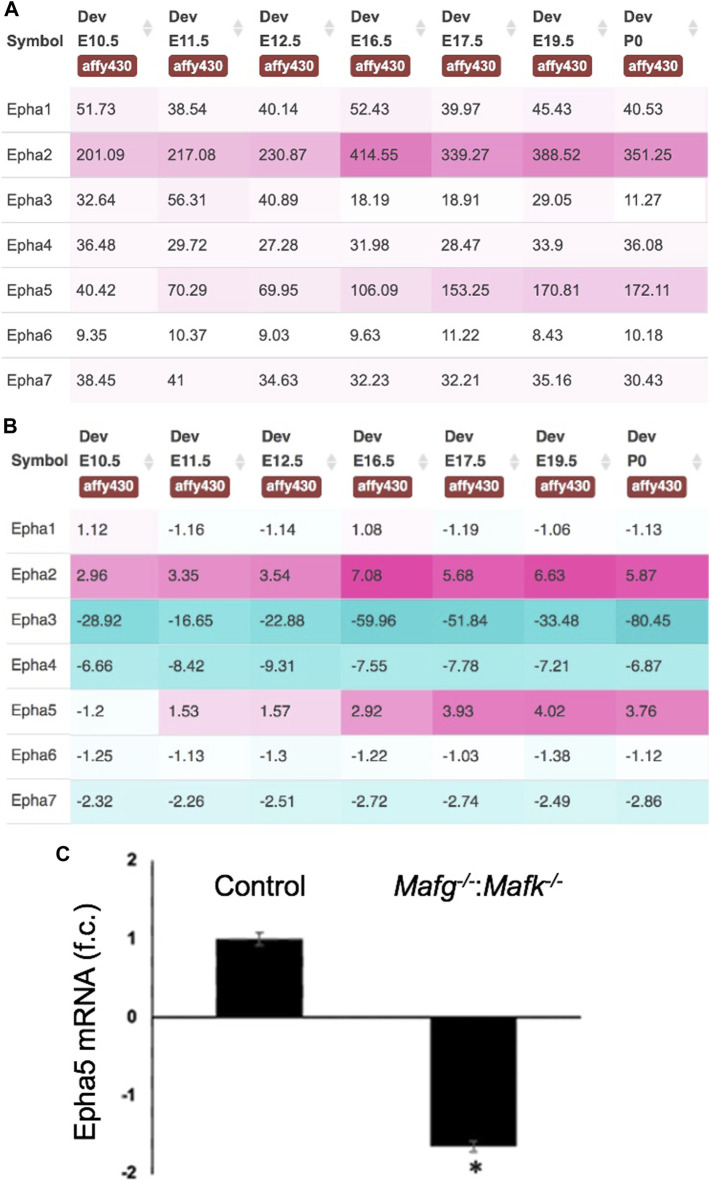
Expression of Eph-signaling receptors in normal lens development and the reduced expression of Epha5 in *Mafg* ^−/−^:*Mafk* ^−/−^ lens. **(A)** iSyTE analysis of various Eph-signaling receptors shows that Epha5, which is identified by RNA-seq analysis among the DEGs in *Mafg* ^−/−^:*Mafk* ^−/−^ lenses, exhibits high expression and **(B)** high lens-enriched expression, second only to Epha2 that is linked to congenital cataract in humans and in animal models. **(C)** RT-qPCR validation of abnormally reduced expression of *Epha5* in *Mafg* ^−/−^:*Mafk* ^−/−^ lenses. Numbers in **(A)** represent normalized microarrays fluorescence intensity units as described in Kakrana and coworkers (2018). Fold-change was calculated using the ΔΔCT-method using *Gapdh* as a house-keeping gene and statistical significance was determined using a two-sample Student’s *t*-test. Asterisk indicates *p* ≤ 0.05.

**FIGURE 9:**
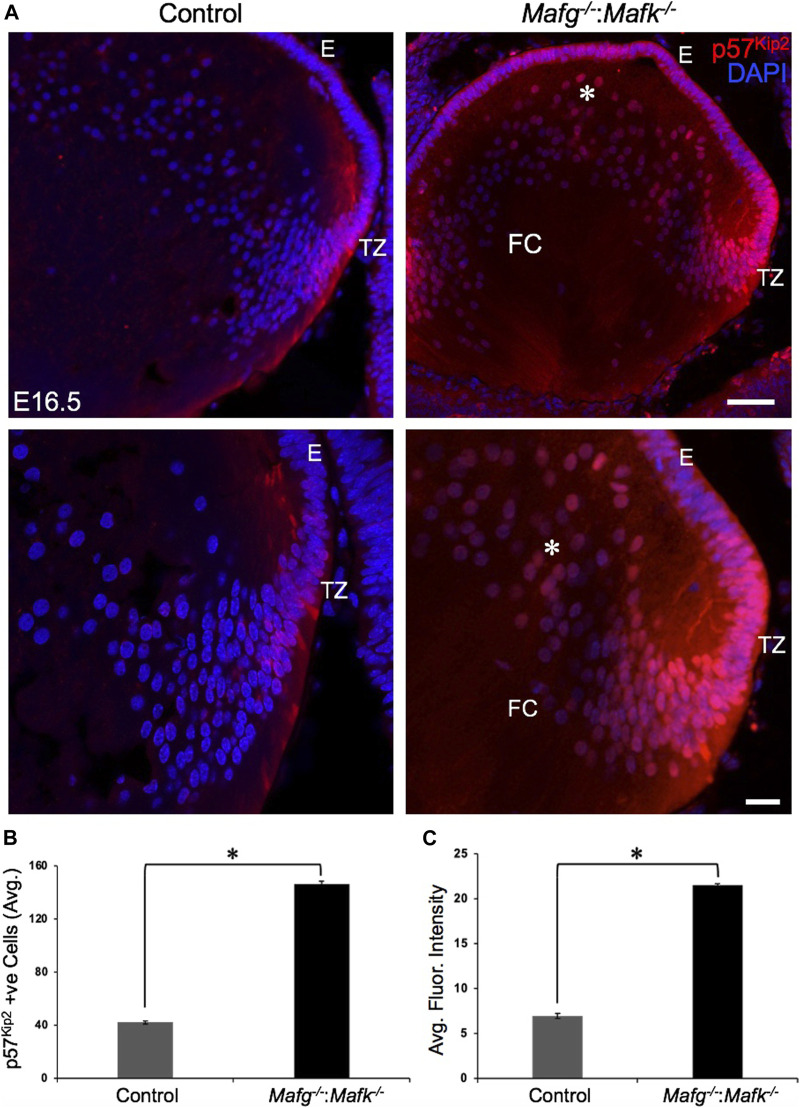
Cdkn1c (p57^Kip2^) protein levels are elevated in *Mafg* ^−/−^:*Mafk* ^−/−^ lens. **(A)** Immunostaining shows Cdkn1c (p57^Kip2^) protein levels are elevated in the transition zone and in fiber cells (asterisk) in E16.5 *Mafg* ^−/−^:*Mafk* ^−/−^ lenses compared to control. **(B)** Quantification of cells from transition zone and fiber compartment exhibiting Cdkn1c (p57^Kip2^) signal in *Mafg* ^−/−^:*Mafk* ^−/−^ lenses compared to control. **(C)** Quantification of average fluorescence intensity of Cdkn1c (p57^Kip2^) signal in *Mafg* ^−/−^:*Mafk* ^−/−^ lenses compared to control. Statistical significance was determined using a two-sample Student’s *t*-test. Abbreviation: E, Epithelium of the lens; FC, Fiber cells of the lens; TZ, Transition zone of the lens. Scale bar for Row 1, 10 μm; Row 2, 8 μm. Asterisk in **(B)** and **(C)** indicates *p* ≤ 0.05.

Next, to further prioritize candidate genes relevant to lens biology so as to uncover the underlying molecular changes associated with *Mafg* ^−/−^:*Mafk* ^−/−^ lens defects, we applied various selection criteria to the list of DEGs identified by RNA-seq. These include gene ontology (GO) and pathway analysis as well as iSyTE analysis, which informs on the expression of candidate genes in normal lens development and its altered expression in various gene perturbation mouse models with lens defects/cataract, and has been shown to be effective in prioritizing key genes and pathways in the lens (Lachke et al., 2011, 2012a, 2012b; Wolf et al., 2013; Manthey et al., 2014; Agrawal et al., 2015; Anand et al., 2015, 2018b, 2021; Audette et al., 2016; Cavalheiro et al., 2017; Patel et al., 2017; Kakrana et al., 2018; Krall et al., 2018; Siddam et al., 2018; Padula et al., 2019; Aryal et al., 2020; Barnum et al., 2020; Choquet et al., 2021). We also considered the potential relevance of the candidate genes to lens biology based on their function described in the published literature. In particular, we prioritized candidates that were starkly altered in *Mafg* ^−/−^:*Mafk* ^−/−^ double KO lenses as opposed to in *Mafg* ^−/−^:*Mafk* ^+/-^ compound KO lenses. GO analysis of *Mafg* ^−/−^:*Mafk* ^−/−^ lens DEGs identified several potentially important categories such as “ephrin receptor signaling pathway”, “extracellular matrix”, “cell projection”, “cell junction”, “response to oxidative stress”, “cyclin-dependent protein serine/threonine kinase activity”, “actin filament binding” and “positive regulation of gene expression”, among others (Figure 5). Based on their differential expression in *Mafg* ^−/−^:*Mafk* ^−/−^ lenses and potential significance to lens development, several candidates from these categories were selected for further analysis by iSyTE and independent validation by RT-qPCR (Table 3). iSyTE analysis showed that majority of these candidates exhibit significant expression or “enriched” expression in normal lens development (Supplementary Figure S7). Further, several of these candidates were found to also be misexpressed in specific gene perturbation mouse models with lens defects or cataract (Supplementary Figure S8). RT-qPCR analysis showed over 10-fold reduction of *Mafg* and *Mafk* transcripts in *Mafg* ^−/−^:*Mafk* ^−/−^ lenses. As reported previously for *Mafg* ^−/−^:*Mafk* ^+/-^ compound KO lenses (Agrawal et al., 2015), RT-qPCR confirmed elevated expression of *Hmox1* and reduced expression of *Trex1* in *Mafg* ^−/−^:*Mafk* ^−/−^ double KO lenses, suggesting that some genes were similarly differentially expressed regardless of whether one or both copies of Mafk were absent in the context of homozygous deletion of Mafg (Figure 6). Importantly, we sought to confirm the differential expression of those genes that were starkly altered only in *Mafg* ^−/−^:*Mafk* ^−/−^ double KO lenses compared to control and *Mafg* ^−/−^:*Mafk* ^+/-^ compound lenses, as these would likely contribute to the early onset lens defects observed only in the double KO animals. Among these candidate genes, the peroxidase enzyme *PXDN*—secreted in the extracellular matrix, identified as lens-enriched by iSyTE and shown to linked to congenital cataract and other ocular defects in human or animal models—was found to be significantly overexpressed in *Mafg* ^−/−^:*Mafk* ^−/−^ lenses (Figure 7). Furthermore, the ephrin signaling receptor, *Epha5*, was found to be significantly reduced in *Mafg* ^−/−^:*Mafk* ^−/−^ double KO lenses. This is interesting because iSyTE analysis shows that Epha5 exhibits enriched expression in the lens, second only to the other ephrin receptor, Epha2, the perturbation of which is linked to cataract in humans and animal models (Figure 8). Moreover, Epha5 is known to be the receptor for the ligand Efna5 (ephrin-A5), the perturbation of which is linked to lens defects (Cheng and Gong, 2011; Cheng et al., 2017) resembling those observed in *Mafg* ^−/−^:*Mafk* ^−/−^ lenses. Additionally, several other genes that may contribute to the lens defects were found to be differentially expressed in *Mafg* ^−/−^:*Mafk* ^−/−^ lenses. These include cyclin-dependent kinase *Cdk1*, leucyl t-RNA synthase *Lars2*, mitogen-activated protein kinase *Map3k12* and signal-induced proliferation protein *Sipal1l*, all exhibiting reduced expression in *Mafg* ^−/−^:*Mafk* ^−/−^ lenses (Figure 6). In contrast, the genes that were found to be sharply elevated in *Mafg* ^−/−^:*Mafk* ^−/−^ lenses were calmodulin-regulated spectrin associated protein *Camsap1*, collagen *Col3a1* and cyclin-dependent kinase inhibitor *Cdkn1c* (p57^Kip2^) (Figure 6). Further, compared to control, E16.5 *Mafg* ^−/−^:*Mafk* ^−/−^ lenses exhibit elevated levels of Cdkn1c (p57^Kip2^) protein in the transition zone and in differentiating fiber cells (Figures 9A,B). Cdkn1c (p57^Kip2^) protein levels were also elevated in fiber cells located deeper in the *Mafg* ^−/−^:*Mafk* ^−/−^ lens tissue, compared to control. Together, misexpression of these genes likely contribute to the lens defects in *Mafg* ^−/−^:*Mafk* ^−/−^ mice. To examine whether increased cell numbers contributed to the multilayered epithelium defects observed in *Mafg* ^−/−^:*Mafk* ^−/−^ lenses, we next performed staining with the established cell proliferation marker Ki67 (Gerdes et al., 1984). Compared to control, *Mafg* ^−/−^:*Mafk* ^−/−^ lenses exhibit elevated number of cells with Ki67 staining and these were restricted to the epithelium region of the lens (Supplementary Figure S9). However, no stark difference was observed in the percent subset of cells that were Ki67 positive between control and *Mafg* ^−/−^:*Mafk* ^−/−^ lenses at E16.5 (data not shown), suggesting that any change in rate of proliferation had ceased by this stage.

**TABLE 3:** Functional significance of select *Mafg* ^−/−^:*Mafk* ^−/−^ differentially expressed candidates.

Gene	Effect in *Mafg* ^−/−^:*Mafk* ^−/−^ lens	Description	Functional significance	References
*Camsap1*	Up	Calmodulin regulated spectrin associated protein 1	Involved in cytoskeletal control	[Jiang et al., 2014; King et al., 2014; Xiang et al. (2016)]
*Cdk1*	Down	Cyclin dependent kinase 1	Involved in cell cycle progression	Santamaría et al. (2007)
*Cdkn1c*	Up	Cyclin dependent kinase inhibitor 1C	Involved in cell cycle inhibition	Zhang et al. (1998)
*Col3a1*	Up	Collagen type III alpha 1 chain	Involved in extracellular matrix control	Kuivaniemi and Tromp, (2019)
*Epha5*	Down	Eph receptor A5 (Tyrosine kinase)	Involved in signaling with Efna5 (ephrin-A5 ligand) to control cell-cell contract mediated events	Akaneya et al. (2010)
*Hmox1*	Up	Heme oxygenase	Involved in oxidative stress response	Zheng et al. (2010)
*Lars2*	Down	Mitochondrial leucyl-tRNA synthetase	Involved in mitochondrial protein synthesis; homolog associated with autophagy	Pierce et al. (2013)
*Map3k12*	Down	Mitogen-activated protein kinase kinase 12 (Leucine-zipper domain and serine/threonine protein kinase)	Involved in cell cycle control and differentiation	[Robitaille et al., 2005, Robitaille et al., 2010; Daviau et al. (2011)]
*Pxdn*	Up	Peroxidasin	Involved in extracellular matrix formation	[Khan et al., 2011; Yan et al. (2014)]
*Sipa1l1*	Down	Signal induced proliferation associated 1 like 1	Potentially involved in actin cytoskeleton organization	[Pak et al., 2001; Matsuura et al. (2021)]
*Trex1*	Down	Three prime repair exonuclease 1	Potentially involved in DNA repair	Yang et al. (2007)

## Discussion

**FIGURE 10:**
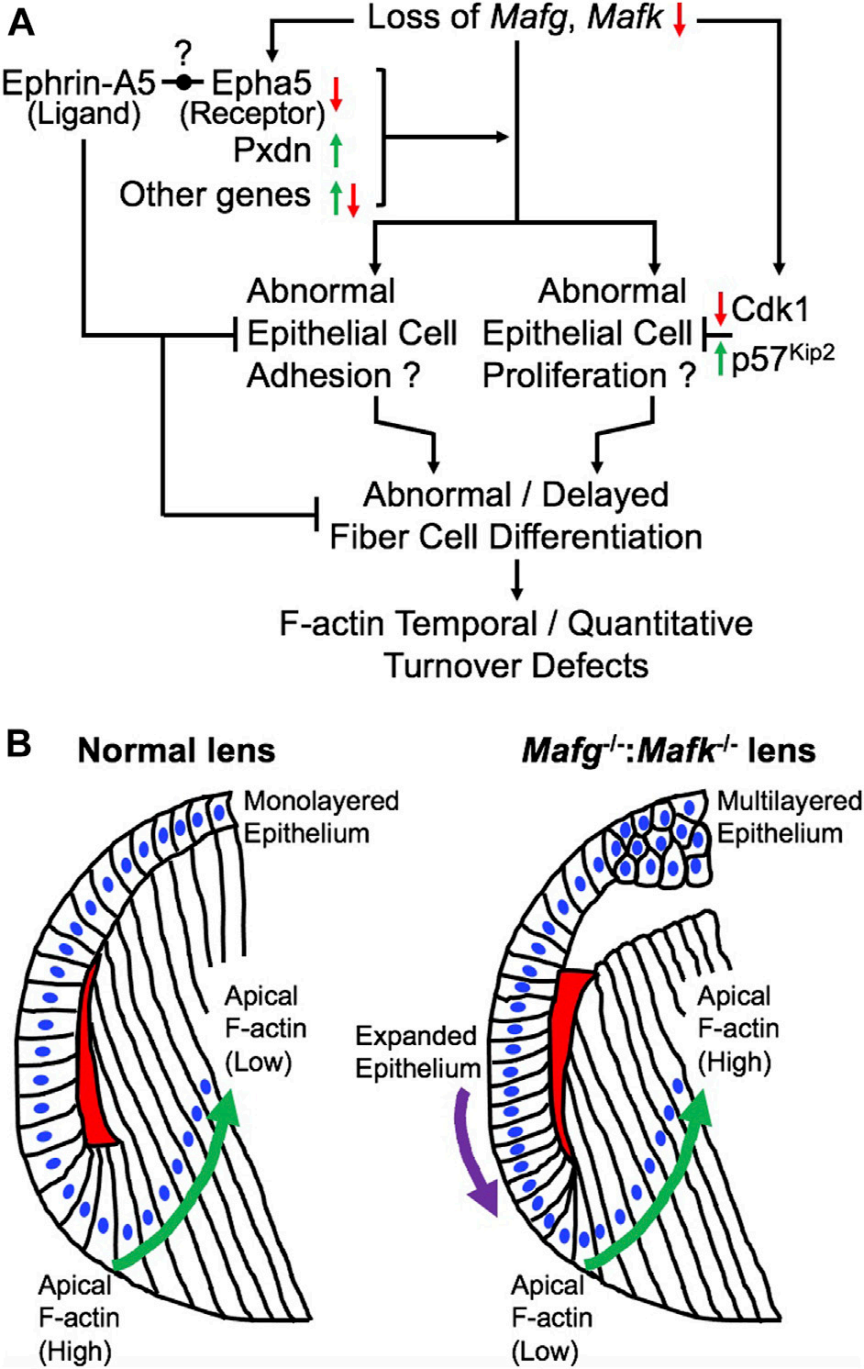
Model for the role of Mafg and Mafk in embryonic lens development. **(A)** Based on the findings in this report, a model is proposed for the role of Mafg and Mafk in lens development. Loss of Mafg and Mafk results in misexpression of several genes, including the cataract-linked extracellular matrix peroxidase enzyme Pxdn and Eph-signaling receptor Epha5. Interestingly, previous work has shown that loss of an ephrin ligand, Efna5 (Ephrin-A5), causes similar lens defects. It was recently proposed by Cheng and co-workers (2017) that an Eph receptor—other that Epha2—may be involved in interactions with Efna5 (Ephrin-A5). Based on these new data, it is tempting to propose Epha5 could represent this receptor. Further, it can be speculated that correct dosage of the cataract-linked gene Pxdn, which is involved in extracellular matrix organization and which is also involved in cell proliferation, may be necessary for proper lens epithelium formation, and its over or under-expression results in lens defects, thus explaining the recruitment of Mafg and Mafk to ensure correct dosage of Pxdn in the lens. These, together with misexpression of other genes result in abnormal epithelial cell adhesion in the lens. It is possible that reduced expression of Cdk1 (positive regulator of the cell cycle) and the elevated expression of p57^Kip2^ (negative regulator of the cell cycle) may reflect perturbations in the cell cycle in *Mafg* ^−/−^:*Mafk* ^−/−^ lenses. Together these epithelial defects may contribute to abnormal or delayed fiber cell differentiation that impacts the optimal distribution of F-actin in these cells. **(B)** In normal lens, the epithelium is maintained as a uniform monolayer. Epithelial cells exit the cell cycle in the transition zone that commence differentiation into fiber cells. In early stages of fiber cell differentiation, F-actin (indicated by red) in the apical region of the cells is observed to be high (Note: the red region is a schematic representative of the overall F-actin staining pattern in this region and further cellular details, such as how cells form the fulcrum region, are not shown). As cells progress in the differentiation program (indicated by green arrow), F-actin levels in their apical regions get reduced. In contrast, *Mafg* ^−/−^:*Mafk* ^−/−^ lenses exhibit loss of a uniformed, monolayer of epithelium (indicated by purple arrow). Further, in *Mafg* ^−/−^:*Mafk* ^−/−^ lenses, F-actin levels are low in the apical region of fiber cells in early stages of differentiation. In contrast to normal lenses, F-actin levels in the apical regions are not observed to be reduced as cells progress in the differentiation program. It will be important in future studies to examine how the crosstalk between small Mafs and their downstream targets control these cell differentiation events.

Previous work showed that loss of the small Maf transcription factors Mafg and Mafk, either individually, or in different allelic combinations result in distinct cellular defects (Blank, 2008; Kannan et al., 2012; Katsuoka and Yamamoto, 2016). However, the role of small Mafs in embryonic lens development remained unaddressed. In an earlier study, we demonstrated that removal of two copies of *Mafg* and one copy of *Mafk* cause lens defects late in life resulting in cataract (Agrawal et al., 2015). Here, we report that mice carrying homozygous deletion of *Mafg* and *Mafk* exhibit embryonic lens defects. We find that *Mafg* ^−/−^:*Mafk* ^−/−^ lens defects are detectable at E12.5 that get progressively severe, such that, by E16.5, the epithelium appears multilayered and there is abnormal accumulation of F-actin in early differentiating fiber cells that appear disorganized. We took an unbiased approach to gain insights into the molecular basis of these defects and performed high-throughput RNA-seq on *Mafg* ^−/−^:*Mafk* ^−/−^ lenses. GO analysis of *Mafg* ^−/−^:*Mafk* ^−/−^ lens DEGs identified several candidates in distinct pathways, involved in cell proliferation, extracellular matrix, cell junction and cytoskeleton control, whose misregulation can help explain the cellular basis of the lens defects, as described in the proposed model (Figure 10). These include the extracellular matrix heme peroxidase Pxdn, mutations in which are linked to human ocular defects, including cataract (Khan et al., 2011; Yan et al., 2014). Pxdn is found to be elevated in several types of cancer and is associated with its poor prognosis and indeed, its overexpression is involved in promoting cell proliferation (Zheng and Liang, 2018). Further, co-expression of Pxdn along with Hmox1, which is also found to be elevated in *Mafg* ^−/−^:*Mafk* ^−/−^ lens, is thought to promote cell proliferation (Tauber et al., 2010). Interestingly, Pxdn has recently been recognized to be a target of the TF Nrf2 (Hanmer and Mavri-Damelin, 2018), which is an established partner protein of small Mafs, binding together of which impact their downstream control over target genes (Katsuoka et al., 2005). Further, Nrf2 deficiency is associated with lens defects and cataract (Rowan et al., 2021). Thus, overexpression of Pxdn, resulting from Mafg, Mafk deletion, may impact cell proliferation and altered ECM, which together contribute to the epithelial defects observed in *Mafg* ^−/−^:*Mafk* ^−/−^ lens. It can be further postulated that the recruitment of Mafg and Mafk for regulation of Pxdn in the lens suggest that multiple pathways may converge to ensure optimal levels of this important protein in the lens.

In addition to Pxdn, an eph signaling receptor, Epha5, was found to be significantly downregulated in *Mafg* ^−/−^:*Mafk* ^−/−^ lens. It has been shown that loss of an established ligand—Ephrin A5 (also known as Efna5)—results in lens defects and cataract in mouse (Cooper et al., 2008; Son et al., 2013; Biswas et al., 2016). Further, the Eph signaling receptor, Epha2, is known to be involved in lens development and cataract (Shiels et al., 2008; Jun et al., 2009; Cheng and Gong, 2011; Shi et al., 2012; Cheng et al., 2013). The lens defects observed in the knockout models of these Eph pathway genes—albeit influenced by the specific mouse strains in which the experiments were carried out on—overlap with the lens defects observed in *Mafg* ^−/−^:*Mafk* ^−/−^ lens. Interestingly, careful studies of Epha2 and Ephrin-A5 double knockout mouse lenses (Cheng et al., 2017; Zhou and Shiels, 2018; Murugan and Cheng, 2022) have suggested that additional Eph signaling receptors, besides Epha2, may be involved in lens development. Our iSyTE analysis shows that Epha5 is the second most abundant Eph signaling receptor—after Epha2—in embryonic lens development. Thus, our observations of high lens-enriched expression of Epha5, along with its reduction in *Mafg* ^−/−^:*Mafk* ^−/−^ lens, which exhibit epithelial and fiber defects that overlap with those observed in Ephrin A5 (Efna5) ligand knockout mice, together present a provocative hypothesis that Epha5 may be an additional receptor involved in Eph signaling in the lens—which can be tested in future studies. The potential relevance of Epha5 downregulation upon Mafg, Mafk KO mice, in different KO allelic combinations, can also be explored further in the context of neurological defects observed in these animals.

Misexpression of other downstream targets of Mafg and Mafk may also contribute to the defects observed in *Mafg* ^−/−^:*Mafk* ^−/−^ lens. For example, based on its known role in other cells (Pak et al., 2001; Matsuura et al., 2021), Sipa1l1 may be involved in regulation of the actin cytoskeleton, and its reduced expression may contribute to the cytoskeletal defects in *Mafg* ^−/−^:*Mafk* ^−/−^ lens. Interestingly, Sipa1l1 is found to be in the eph receptor signaling pathway according to GO analysis. Similarly, misexpression of Camsap1, known to be involved in cytoskeletal control by binding to spectrin (Jiang et al., 2014; King et al., 2014; Xiang et al., 2016), may contribute to the observed lens defects. Further, overexpression of the collagen encoding gene *Col3a1* in *Mafg* ^−/−^:*Mafk* ^−/−^ may result in ECM defects and thus contribute to the lens defects (Kuivaniemi and Tromp, 2019).

Dimerization of small Maf transcription factors with other bZIP family members are known to control their specific function. Interestingly, we find a leucine zipper containing mitogen-activated serine/threonine protein kinase, *Map3k12*, to have significantly reduced expression in *Mafg* ^−/−^:*Mafk* ^−/−^ lens. Map3k12 is potentially involved in cell differentiation and control of the cell cycle (Robitaille et al., 2005, 2010; Daviau et al., 2011). Thus, its reduced expression may contribute to the lens defects in *Mafg* ^−/−^:*Mafk* ^−/−^ lens. The proposed model outline the various downstream events that help provide a molecular and cellular explanation for understanding the pathology observed in *Mafg* ^−/−^:*Mafk* ^−/−^ mouse lenses (Figure 10). In particular, it is interesting to note that in *Mafg* ^−/−^:*Mafk* ^−/−^ lenses, F-actin levels are low in the apical region of fiber cells in early stages of differentiation and are not reduced as cells progress in the differentiation program. This suggests that Mafg and Mafk contribute to the spatiotemporal control over F-actin deposition and/or turnover in early lens fiber differentiation (Figure 10). It will be interesting to explore, in future, the potential crosstalk of Mafg, Mafk and Eph-signaling, as well as other factors (e.g., Pxdn) in regulating these cytoskeletal events in the lens. Other finer aspects of the *Mafg* ^−/−^:*Mafk* ^−/−^ lens defects, such as the abnormal “dip-like” appearance in the central epithelium, may be a result of diverse—not necessarily mutually exclusive—events (e.g., secondary to abnormal epithelial cell activity or change in extracellular matrix proteins or as an indirect result due to fiber cells not expanding optimally to accommodate epithelial cells as a monolayer, etc.) that are areas that can be characterized in the future. Also, it should be noted that in the present study RNA-seq was performed on whole lens tissue and therefore subtle changes in gene expression that occur specifically in epithelial or fiber cells may not be detected. Therefore, future RNA-seq studies on isolated epithelium or fiber cells using laser capture microdissection (LCM) or on single cells may provide new molecular insights into these defects. In sum, these data suggest new directions wherein it can be examined whether these regulatory relationships between small Mafs and their downstream mis-expressed genes, especially those involving Eph-signaling, extracellular matrix proteins and cell cycle regulators, are present in the development and/or homeostasis of other tissues.

## Data Availability

The datasets presented in this study can be found in online repositories (Gene expression omnibus). “Accession Number: GSE207853”.
