# Supplementary material for: Deficiency of the bZIP transcription factors Mafg and Mafk causes misexpression of genes in distinct pathways and results in lens embryonic developmental defects
Source: Front Cell Dev Biol. 2022 Aug 26;10:981893. doi: 10.3389/fcell.2022.981893 (PMC9459095; doi:10.3389/fcell.2022.981893)
Supplement: Supplementary file 1 [file DataSheet1.PDF]

frontiers

Supplementary Material

Supplementary Figures

A

	E10.5 affy430	E11.5 affy430	E12.5 affy430	E16.5 affy430	E17.5 affy430	E19.5 affy430	P0 affy430
Mafg	614.4	649.11	1048.97	1101.87	1056.77	1015.04	953.21
Mafk	205.34	196.92	219.86	246.56	274.64	245.29	294.85

B

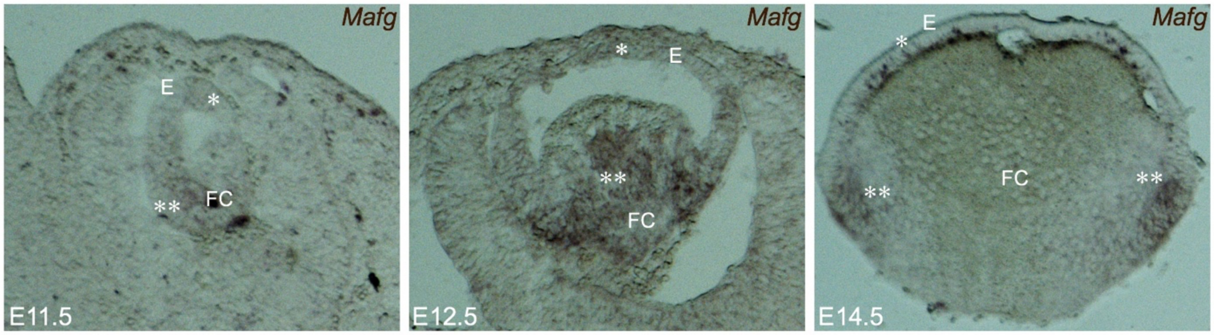

C

	E14.5		E16.5		E18.5		P0	
	Epi.	Fiber	Epi.	Fiber	Epi.	Fiber	Epi.	Fiber
Mafg	709	2268	878	1820	567	1544	660	1290
Mafk	74	72	97	127	79	146	111	142

Supplementary Figure 1. iSyTE analysis demonstrates that *Mafg* and *Mafk* transcripts are expressed in the mouse lens. (A) *Mafg* and *Mafk* transcripts are expressed in the mouse lens embryonic development from early stages (embryonic day (E)10.5) onward. Numbers represent normalized microarrays fluorescence intensity units as described in Kakrana and coworkers (2018). **(B)** *In situ* hybridization shows Mafg expression at E11.5, E12.5 and E14.5 in the epithelium (*) and fiber cells (**). Abbreviation: E, Epithelium of the lens; FC, Fiber cells of the lens. **(C)** Data from RNA-seq analysis on isolated epithelium and fiber cells from different mouse embryonic lens stages (number represent normalized gene-expression as FPKM (Fragments Per Kilobase of transcript per Million mapped reads), as described in Zhao and coworkers (2018)). Row-specific heat-map red-green gradation represent expression of Mafg or Mafk in lens epithelium and fiber cells at different mouse developmental stages.

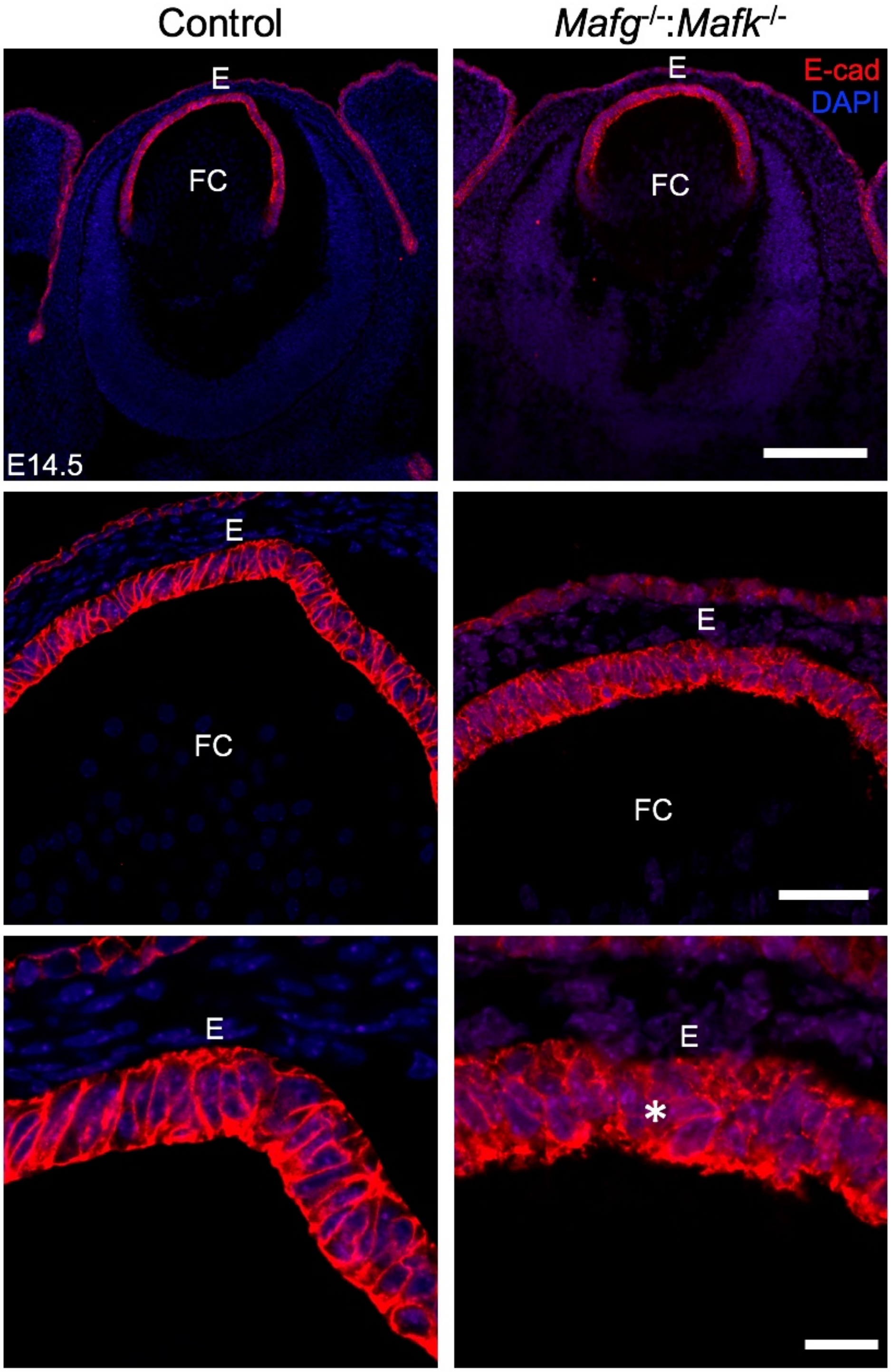

Supplementary Figure 2. E-cadherin expression appears abnormal in *Mafg*$^{-/-}$:*Mafk*$^{-/-}$ lens epithelium at E14.5. Immunostaining for E-Cadherin and co-staining with DAPI shows that at stage E14.5, compared to control, *Mafg*$^{-/-}$:*Mafk*$^{-/-}$ lens exhibits regions of disorganization in the epithelium (asterisk). Abbreviation: E, Epithelium of the lens; FC, Fiber cells of the lens. Scale bar for Row 1, 20 μm; Row 2, 10 μm; Row 3, 8 μm.

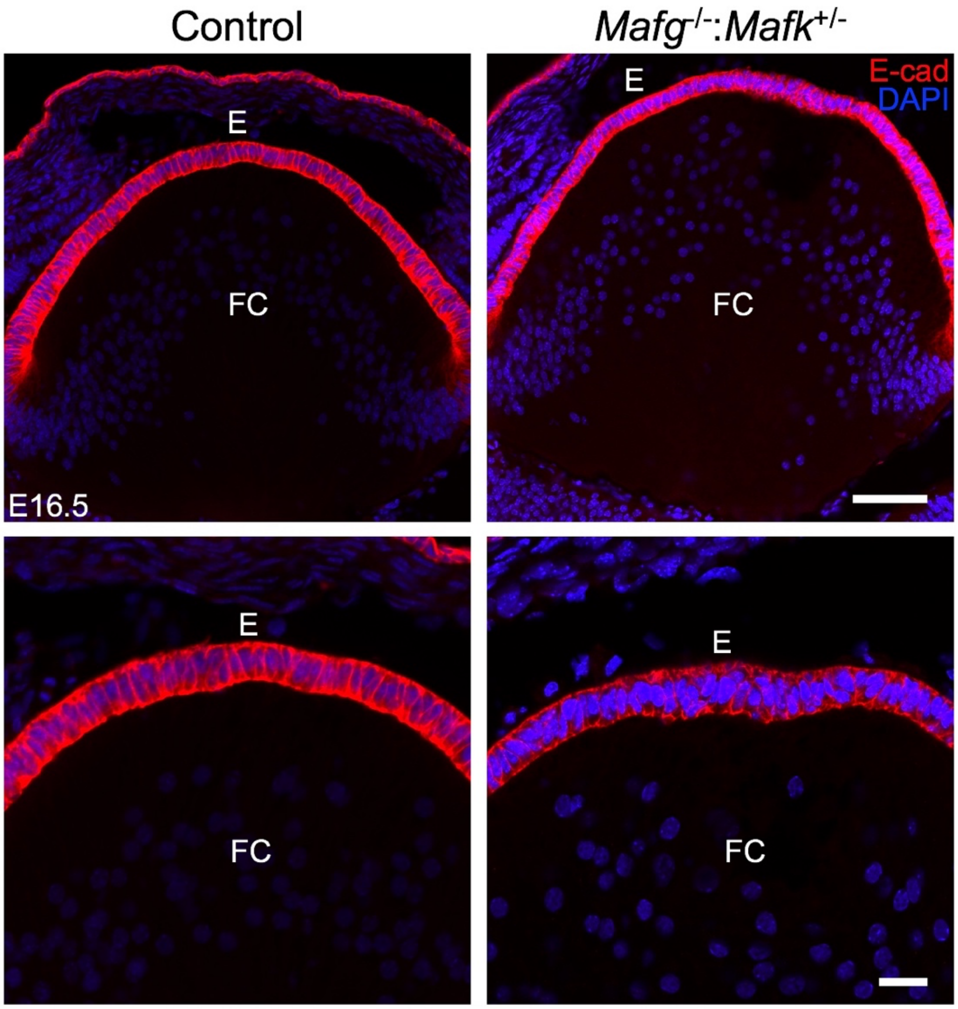

Supplementary Figure 3. *Mafg*$^{-/-}$:*Maf* $^{+/-}$ compound KO mice do not exhibit severe lens epithelial cell defects at E16.5. Immunostaining for E-Cadherin and co-staining with DAPI shows that at stage E16.5, *Mafg*$^{-/-}$:*Mafk* $^{+/-}$ compound KO lenses do not exhibit severe epithelial cells compared to those observed in *Mafg*$^{-/-}$:*Mafk*$^{-/-}$ lens. Abbreviation: E, Epithelium of the lens; FC, Fiber cells of the lens. Scale bar for Row 1, 20 µm; Row 2, 10 µm.

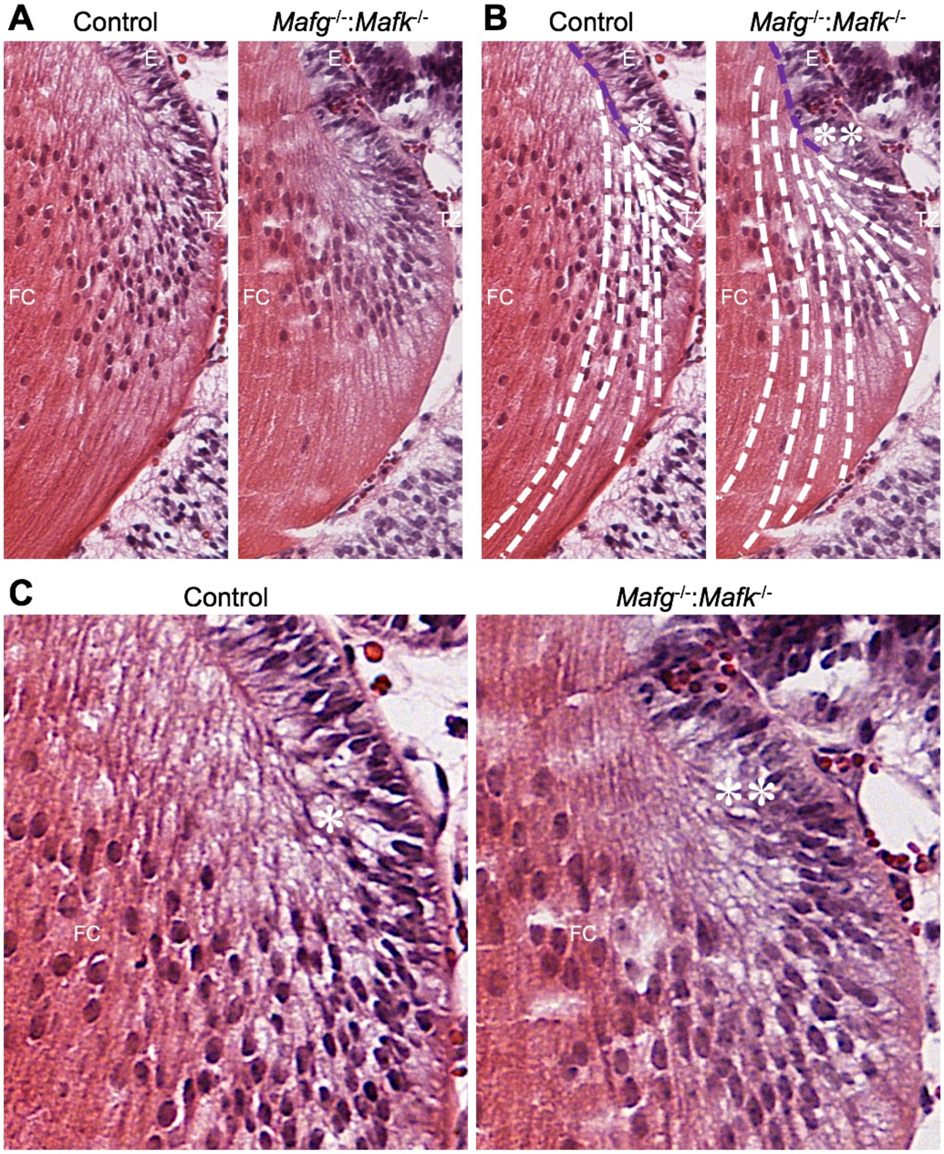

Supplementary Figure 4. Histological analysis of lens defects in *Mafg*$^{-/-}$:*Mafk*$^{-/-}$ mice. (A) Histology shows that in contrast to control, lenses from E16.5 *Mafg*$^{-/-}$:*Mafk*$^{-/-}$ mice appear abnormal. **(B)** Tracing of cells (white dashed line) shows that control lenses exhibit differentiating cells to converse on the fulcrum region (asterisk) as expected. In contrast, this pattern appears abnormal near and beyond the fulcrum region (two asterisks) in lenses of *Mafg*$^{-/-}$:*Mafk*$^{-/-}$ mice. Tracing by purple dashed line indicates a smooth curve between epithelium and fiber cells in control, while this appears to be abnormal in lenses of *Mafg*$^{-/-}$:*Mafk*$^{-/-}$ mice. While more mature fiber cells in control appear to curve "inwards" in the same direction, generally following the curvature of the lens, fiber cells in *Mafg*$^{-/-}$:*Mafk*$^{-/-}$ mouse lens appear to not follow such a pattern. **(C)** The fulcrum region between epithelium and fiber appears tight (asterisk) in the control while it appears less tight or more "diffused" (two asterisks) in *Mafg*$^{-/-}$:*Mafk*$^{-/-}$ mouse lens. Abbreviation: E, Epithelium of the lens; FC, Fiber cells of the lens; TZ, Transition zone of the lens.

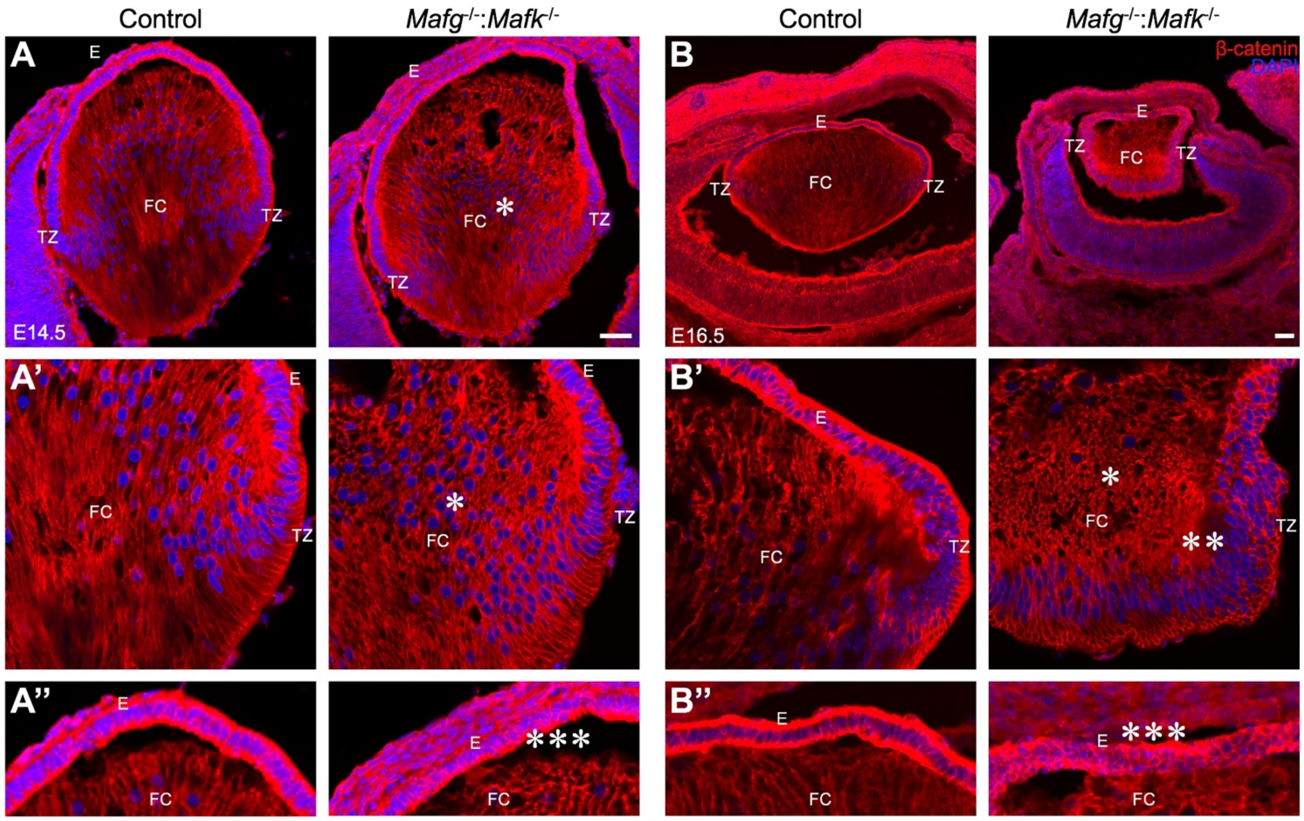

Supplementary Figure 5. Immunostaining for β-catenin serves to demonstrate lens defects in *Mafg*$^{-/-}$:*Mafk*$^{-/-}$ mice. **(A)** Immunostaining for β-catenin and co-staining with DAPI shows that E14.5 and **(B)** E16.5 *Mafg*$^{-/-}$:*Mafk*$^{-/-}$ mice exhibit non-uniform arrangement of epithelium and fiber cells (indicated by asterisk) compared to control at their age matched controls, which is also observed at **(A', B')** higher magnification. In addition to fiber cells (one asterisk), the transition zone appears to be expanded and deeper into the fiber cell region (indicated by two asterisks) in E16.5 *Mafg*$^{-/-}$:*Mafk*$^{-/-}$ lenses (B'). **(A'')** Defects in the epithelium architecture are observed at E14.5 (indicated by three asterisks) that are more severe **(B'')** at E16.4 (indicated by three asterisks). Abbreviation: E, Epithelium of the lens; FC, Fiber cells of the lens; TZ, Transition zone of the lens. Scale bar, 10 μm.

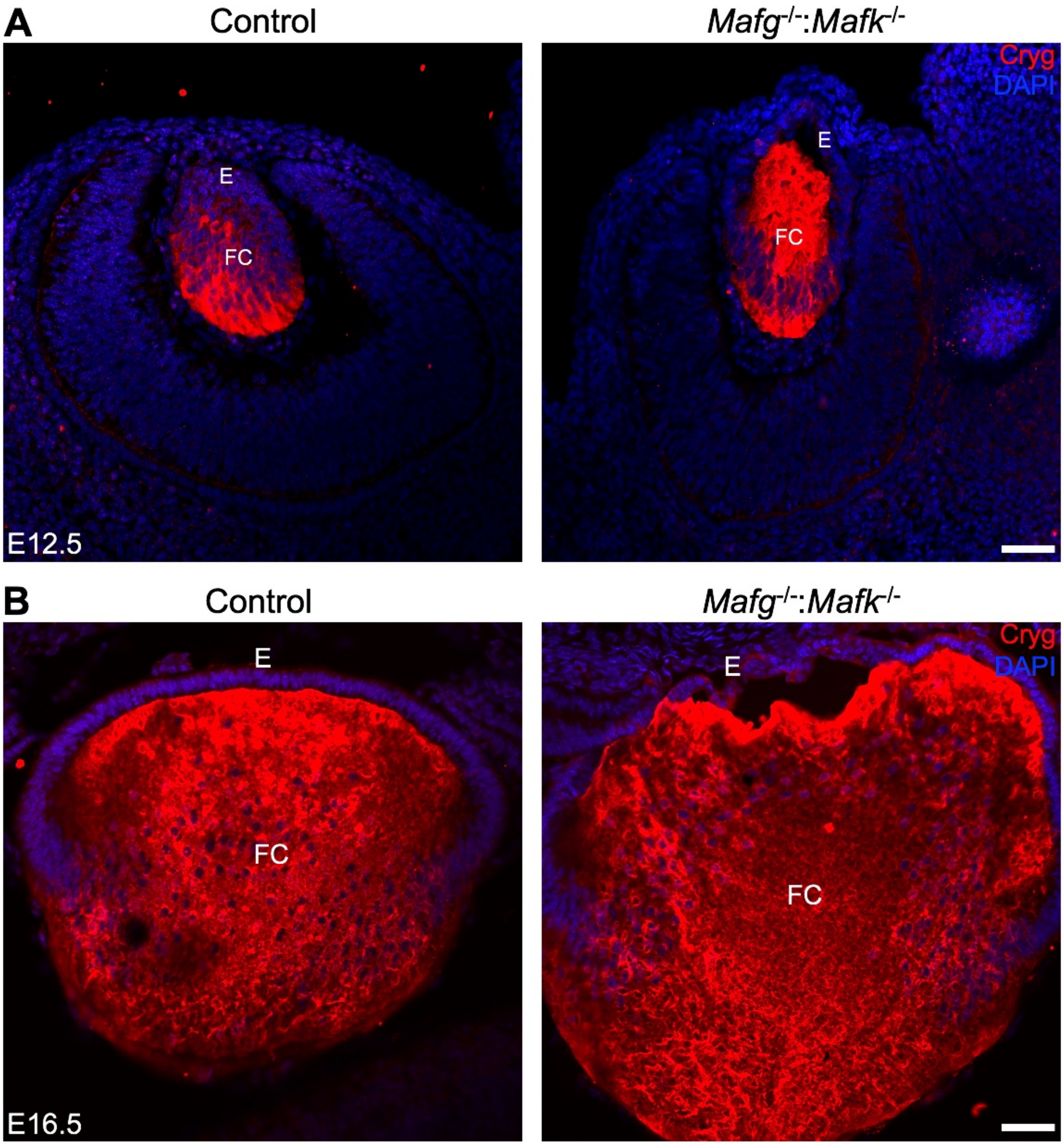

Supplementary Figure 6. Immunostaining shows that gamma cyrstallin expression is unaltered in *Mafg*$^{-/-}$:*Mafk*$^{-/-}$ mice. (A) Immunostaining for gamma crystallin and co-staining with DAPI shows that at stages E12.5 and **(B)** E16.5, *Mafg*$^{-/-}$:*Mafk*$^{-/-}$ lenses do not exhibit defects in its abundance or expression pattern. Abbreviation: E, Epithelium of the lens; FC, Fiber cells of the lens. Scale bar, 20 µm.

	Dev E10.5 affy430	Dev E11.5 affy430	Dev E12.5 affy430	Dev E16.5 affy430	Dev E17.5 affy430	Dev E19.5 affy430	Dev P0 affy430
Camsap1	550.19	466.76	406	396.57	431.52	385.66	429.15
Cdk1	2832.19	1673.18	977.09	498.74	418.16	493.08	156.19
Cdkn1c	2319.29	4893.52	6558.22	6288.84	6628.95	6400.5	6247.69
Col3a1	441.05	1193.56	1171.42	123.65	64.91	138.25	65.55
Hmox1	128.53	122.94	159.65	1588.14	2242.94	403.28	4212.38
Lars2	304.98	304.78	260.84	250.78	202.25	112.52	212.53
Map3k12	140.88	162.32	156.19	168.58	161.06	150.55	143.02
Sipa1l1	241.12	222.9	278.68	476.31	417.57	487.96	402.25

Supplementary Figure 7. iSyTE analysis of select differentially expressed genes in *Mafg*$^{-/-}$:*Mafk*$^{-/-}$ mouse lens. iSyTE analysis shows that prioritized gene candidates are significantly expressed in normal mouse lens development and therefore may be relevant to lens biology. Numbers represent normalized microarrays fluorescence intensity units as described in Kakrana and coworkers (2018).

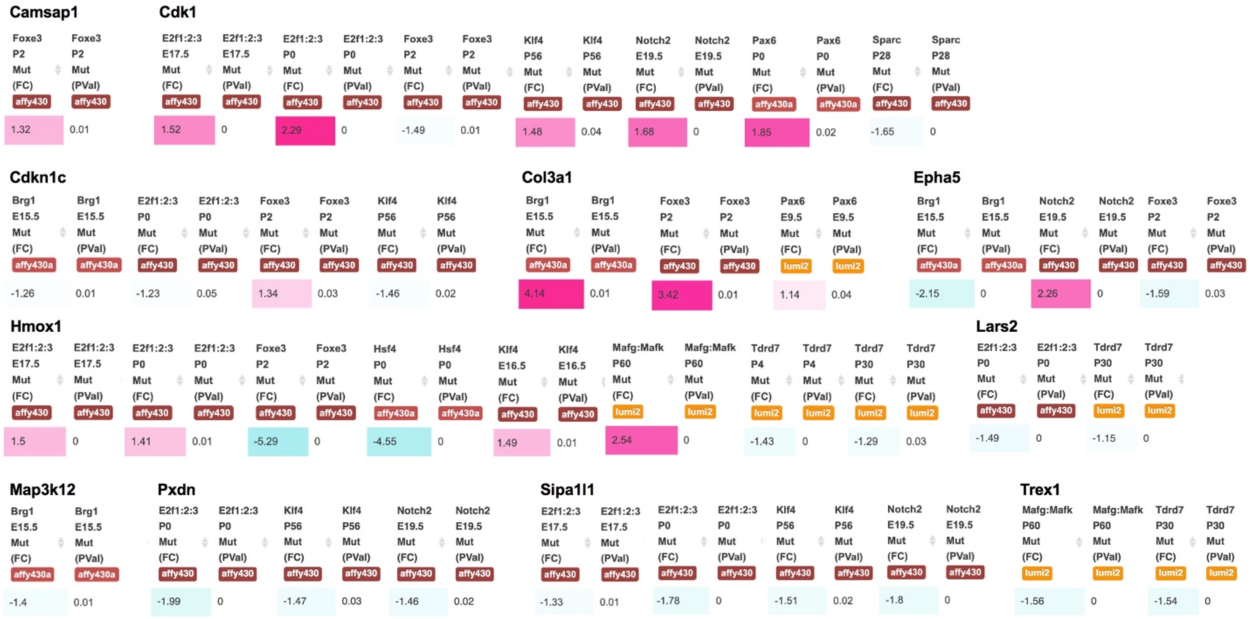

Supplementary Figure 8. iSyTE analysis of *Mafg*$^{-/-}$:*Mafk*$^{-/-}$ differentially expressed genes in other gene perturbation mouse models exhibiting lens defects/cataract. iSyTE analysis shows that all of the high-priority genes that are differentially expressed in *Mafg*$^{-/-}$:*Mafk*$^{-/-}$ lenses are also found to be misexpressed in the lens in other mouse gene-perturbation models. Expression analysis is shown for *Camsap1*, *Cdk1*, *Cdkn1c*, *Col3a1*, *Epha5*, *Hmox1*, *Lars2*, *Map3k12*, *Pxdn*, *Sipa1l1* and *Trex1* in lens expression datasets (Affymetrix or Illumina microarray platforms) of ten different mouse models that exhibit lens defects and/or cataract. Gene expression changes are in fold-change at $p \leq 0.05$ in the specific gene perturbation mouse model lens compared to control.

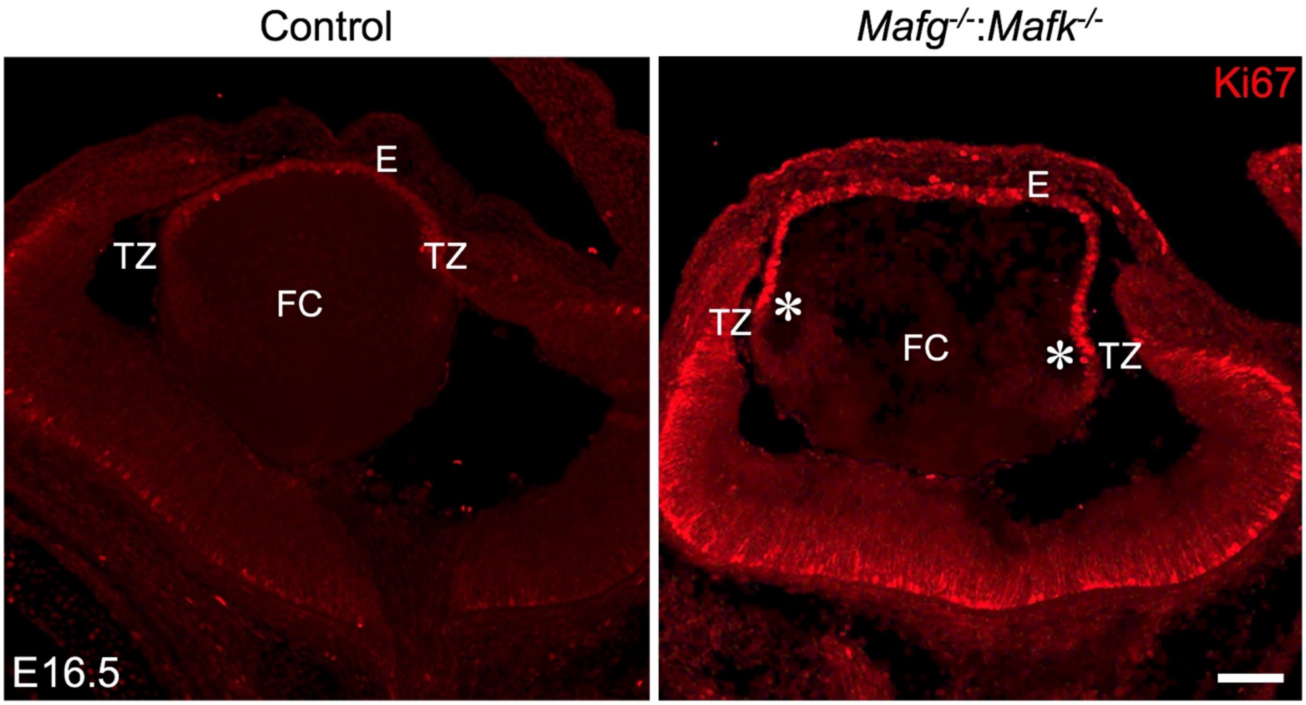

Supplementary Figure 9. Immunostaining shows that compared to control, *Mafg*$^{-/-}$:*Mafk*$^{-/-}$ mice appear to have a higher number of Ki67 positive cells that lie in the lens epithelium at E16.5. Note also that *Mafg*$^{-/-}$:*Mafk*$^{-/-}$ mice show elevated Ki67 fluorescence signal (asterisk). However, there is no apparent difference in the ratio of Ki67 positive nuclei over total number of nuclei between control and *Mafg*$^{-/-}$:*Mafk*$^{-/-}$ mice. Abbreviation: E, Epithelium of the lens; FC, Fiber cells of the lens; TZ, Transition zone of the lens. Scale bar, 10 µm.
